# Energy–Biosensor Synergy: Intrinsic Catalytic Reactions as Label-Free Signal Pathways

**DOI:** 10.3390/s26041205

**Published:** 2026-02-12

**Authors:** Seyyed Mehdi Khoshfetrat, Samaneh Mirsian, Amirreza Khodadadian, Wolfgang Hilber, Clemens Heitzinger

**Affiliations:** 1 Department of Chemistry, Faculty of Basic Sciences, Ayatollah Boroujerdi University, Boroujerd 69199-69737, Iran; 2 Biosensor and Energy Research Center, Ayatollah Boroujerdi University, Boroujerd 69199-69737, Iran; 3 Institute of Microelectronics and Microsensors, Johannes Kepler University Linz, 4040 Linz, Austria; samaneh.mirsian@jku.at (S.M.); wolfgang.hilber@jku.at (W.H.); 4 School of Computer Science and Mathematics, Keele University, Staffordshire ST5 5BG, UK; 5 Machine Learning Unit, TU Wien, 1040 Vienna, Austria; clemens.heitzinger@tuwien.ac.at

**Keywords:** biosensor, redox label, reagentless, hydrogen evolution reaction, oxygen reduction reaction, water splitting

## Abstract

The selection of appropriate signal labels is a central consideration in electrochemical biosensing as it directly determines the achievable detection limits, dynamic range, and overall analytical performance. Conventional electroactive labels require low operating potentials, fast electron-transfer kinetics, and reliable attachment to electrode surfaces or recognition elements. Despite their extensive use, these labels present notable challenges for point-of-care applications, particularly in the detection of small molecules where target binding does not inherently generate a measurable electrochemical output. As a result, most sensing architectures depend on externally added redox reporters, introduced either freely into solution or covalently linked to recognition structures, which increases assay complexity and limits scalability. These limitations have motivated the transition toward energy-based electrochemical signal pathways, such as the hydrogen evolution reaction (HER), oxygen reduction reaction (ORR), and water-splitting reactions. These reactions provide intrinsic electrochemical outputs that eliminate the need for synthetic redox mediators and can operate as built-in catalytic signal sources. Their integration into biosensing platforms simplifies assay design, enhances robustness, and broadens compatibility with diverse target molecules. This review outlines the mechanistic basis connecting HER/ORR/water-splitting reactions to signal generation in biosensors and highlights material design principles that enable their use as reagentless and label-free transduction strategies. Compared with traditional electroactive labels, energy-driven approaches offer simplicity, reduced cost, faster operation, and improved suitability for commercial translation. By establishing a unified framework for energy-based electro-recording mechanisms, this review aims to promote the development of next-generation bioanalytical methods that operate without electroactive labels and expand the applicability of electrochemical biosensing across various domains.

## 1. Introduction

Biosensors are analytical devices used to detect and measure specific biological substances or processes. They typically consist of two fundamental components: a biological recognition element that interacts with the target analyte and a transducer that converts this interaction into a measurable signal such as an electrical, optical, or electrochemical output. A significant challenge in biosensor development is that most biomolecules do not inherently produce a readily detectable signal upon binding. For instance, although antibody–antigen interactions do not generate photons or electrons, they produce only subtle conformational changes, making them difficult to detect directly. As a result, translating biomolecular binding events into clear, selective output signals, especially in complex biological samples containing potentially interfering species, remains difficult.

Upon recognition of biomolecule binding to target molecules, common methods for measuring refractive index, mass, steric bulk, and charge include surface plasmon resonance [1,2,3,4,5,6,7], quartz crystal microbalance [8,9], static microcantilever [10,11,12], impedance spectroscopy [13,14,15], and field-effect transistor (FET) sensors [16,17,18,19]. Among label-free transduction strategies, FET biosensors enable direct electrical readout of biomolecular binding without redox mediators or enzymatic labels. Early silicon nanowire (SiNW) sensors established highly sensitive detection via surface-charge-induced conductance modulation, later extended to sequence-specific DNA sensing and multiplexed biomarker detection [20,21,22]. CMOS-compatible implementations further demonstrated the scalability and practical viability of SiNW-FET immunodetection platforms. Related advances in field-effect biosensing have combined physics-based models, Bayesian inversion, and machine learning to enable quantitative interpretation and rational design of graphene and semiconductor FET sensors [23,24,25,26].

Each of these methods has specific limitations. For instance, surface plasmon resonance, a commercialized adsorption-based approach developed by Biacore [27,28,29], is effective in high-quality buffer samples that are free of interference from other components, leading to false positives in complex real samples. Because all of these proteins have mass, charge, etc., it is difficult, if not impossible, to distinguish between their nonspecific adsorption and binding of the true target [29]. The rapid results, low cost, and ease of use of electrochemical bioassays make them a vital diagnostic tool, especially for point-of-care frameworks, among the detection techniques employed in biosensor designs [30,31,32]. Improvements in detection methods have been the driving force for the development of electrochemical biosensors.

Label-free and label-based electrochemical methods are widely used in various applications and can be differentiated by their underlying operating principles and capabilities. Label-free approaches are generally simple and cost-effective because they detect and quantify analytes without requiring additional chemical tags or markers.

Label-free techniques include certain voltammetric methods and electrochemical impedance spectroscopy (EIS), both of which can detect analytes directly. In contrast, label-based methods rely on the use of specific labels or tags that interact with the target analyte to enhance sensitivity. The use of redox-active molecules as labels is commonly referred to as electrochemical labeling. These label-based strategies are often integrated with other bioanalytical platforms, such as DNA biosensors and immunoassays. Incorporating a label typically improves detection sensitivity, allowing for the measurement of very low analyte concentrations, while the biological recognition element primarily governs selectivity.

In catalytic electrochemical biosensors, signal sensitivity and noise are strongly governed by electron-transfer kinetics and the underlying reaction pathways at the electrode–electrolyte interface [33,34]. Fast and well-defined electron-transfer processes enhance signal-to-noise ratios by producing sharp, reproducible electrochemical responses, whereas sluggish kinetics, multistep pathways, or competing side reactions can introduce background currents, temporal delays, and signal instabilities [23,34]. In particular, catalytic reactions involving multiple electron and proton transfer steps are highly sensitive to surface chemistry, mass transport, and local interfacial environments, all of which influence both amplification efficiency and noise characteristics [35]. Understanding and controlling electron-transfer rates and reaction mechanisms is therefore essential for optimizing sensitivity, minimizing false responses, and achieving reliable signal transduction in label-free and reagent-free catalytic biosensors.

### 1.1. Challenges of Label-Free, Label- and Reagent-Based Electrochemical Detection

#### 1.1.1. Label-Free Electrochemical Techniques

Label-free biosensors can detect the target directly, allowing for rapid analyte detection with little or no sample preparation and enabling real-time evaluations. These types of sensors either rely on the intrinsic electroactivity of nucleic acid molecules in DNA biosensors or are associated with electrochemical changes caused by antibody–antigen interactions or hybridization processes [36]. Since label-free biosensors depend on direct biorecognition events, they require receptors with high specificity and affinity for the target molecule, as well as a sensitive transducer.

To achieve high sensitivity, signal amplification strategies such as the modification of electrodes with high-performance functional materials [37], enzyme-based isothermal amplification [38,39], and single-, dual-, or triple-signal amplification frameworks have been exploited [40,41]. These strategies enhance the detection capabilities of biosensors, making them more effective for identifying low-abundance targets in complex samples. However, to record the electrochemical signal, the use of reagent molecules in the solution or on the electrode surface is often required. For example, [Fe(CN)_6_]^3−/4−^ is a commonly employed negatively charged redox probe in label-free electrochemical biosensors, particularly in EIS, one of the most widely used label-free electrochemical techniques.

EIS-based biosensors monitor the impedance at the electrode/solution interface by imposing a small AC excitation over a constant DC voltage across a frequency range. Label-free electronic signal transduction using impedance biosensors detects target binding through changes in the biorecognition layer at the surface. A solid electrode’s surface develops an electrical double layer when exposed to an electrolyte solution, giving rise to a capacitor-like interfacial behavior. However, achieving signal efficiency in complex biological samples is a challenge for many impedance-based biosensors [34].

In complex biological systems, due to the shielding effect of the electrical double layer at the electrode–electrolyte interface, the effectiveness of signal transduction can be notably reduced. Consequently, the clinical usefulness of the sensors may be limited by reduced sensitivity. Furthermore, with these methodologies, the presence of any electroactive component in the solution has the potential to produce false-positive responses in electrochemical biosensors. These limitations hinder the full potential of label-free electrochemical sensors for personalized healthcare applications.

#### 1.1.2. Label-Based Electrochemical Techniques

Label-based approaches detect a specific analyte in a matrix by using tags or labels [42]. In these assays, reliable and stable electrochemical labels are essential, particularly when the analyte is not electrochemically active. The selection of electrochemically active labels is generally based on two criteria: (i) redox potential and (ii) electron transfer kinetics. High redox potentials or slow electron transfer kinetics can lead to overlap with signals from interfering species in the sample, reducing selectivity and specificity [43,44,45]. Electrochemical labels are commonly divided into three functional categories:

1. Enzyme-type labels responsible for catalyzing specific substrates. Examples include horseradish peroxidase (HRP) and alkaline phosphatase (ALP), which catalyze reactions that produce electroactive products detectable by the sensor. This approach increases sensitivity by generating higher concentrations of detectable species.
2. Labels used as carriers for a high volume of electroactive substrates, increasing sensitivity by releasing or generating more detectable species [46,47].
3. Labels that are intrinsically electrochemically active materials. Examples include metal nanoparticles, quantum dots, and other nanomaterials with strong and stable redox properties.

Enzymes produce redox-active molecules, which are commonly applied as bioassay labels. By providing a high number of detectable redox substances per label, enzymes contribute to lowering the limit of detection (LOD) in biosensing applications. However, enzymes can be delicate and susceptible to environmental factors such as pH and temperature. The detection time in enzyme-based assays is determined by the enzymatic turnover rate, which must be carefully controlled to ensure accurate and timely detection [48].

The utilization of compounds as carriers for electroactive materials is important in electrochemical methods. These carriers facilitate the efficient transport and interaction of electroactive components within the sensing system. In electrochemical sensors and supercapacitors, carrier compounds enable effective transport and high loading of redox labels, thereby enhancing overall device performance. The appropriate selection of carrier compounds is essential, as it can influence conductivity, stability, and label-loading capacity. Ongoing research continues to focus on developing improved carrier materials with enhanced properties to advance biosensor technologies and related fields. Based on the information presented thus far, the loading capacity of labels is regarded as the primary determinant of a carrier’s functionality. This is why nanoparticles play a particularly important role within this category. Their high surface-area-to-volume ratio allows efficient loading of electroactive materials, leading to enhanced sensitivity and performance in electrochemical devices.

Taking advantage of nanoparticles’ high surface area as carriers, increasing the loading number of redox labels on these nanoparticles can help overcome the low sensitivity of electroactive compounds. However, weak π–π interactions and the need for covalent attachment of redox labels on carrier nanoparticles lead to a limited loading capacity [49].

Alternatively, the high porosity and tunable pore sizes of metal–organic frameworks (MOFs) provide a suitable supporting matrix for incorporating high quantities of redox species inside the MOF structure [50,51,52,53,54,55,56,57]. However, this amplification alone is not sufficient for trace bioanalysis [58]. In addition, low conductivity and MOF instability in aqueous solutions impose further limitations on their use in electrochemical biosensors [59,60,61].

In another approach, electroactive labels such as nanoparticles and organic compounds are directly employed for detection by exploiting their inherent redox properties. The signal is substantially enhanced because each nanoparticle label contains large numbers of oxidizable or reducible electroactive centers. Increasing numbers of electrochemical immuno/biosensor designs have used biomolecules modified with redox-active reporters. Despite improved detection limits and the elimination of additional tags or redox indicators, challenges remain when using high-potential redox species or aggressive reagents during detection. For example, strong acids (such as HCl [62] or ${\mathrm{HNO}}_{3}$ [47,63]) or powerful oxidizers (such as ${\mathrm{Br}}_{2}$ [64], ${\mathrm{KMnO}}_{4}$ [65,66], and ${\mathrm{OCl}}^{-}$ [67]) may be required, increasing protocol complexity and reducing specificity and selectivity. Balancing enhanced sensitivity with safety, operational simplicity, and specificity is therefore crucial in biosensor design.

When considering organic materials, it is important to note that the redox center of many organic components is typically present in a 1:1 ratio with the analyte. This lack of intrinsic amplification can limit their utility in practical diagnostic devices, although exceptions exist. Furthermore, the performance of electrochemically active organic labels in complex environments is another challenge [68]. For example, while a sensor using the reporter methylene blue (MB) shows good stability in serum-containing media, other modified electrodes under similar conditions may exhibit a significant drop in current and lose around 50% of their original signal. This variability highlights the difficulty of achieving consistent and reliable performance for organic-based electrochemical labels in diverse biological environments. Efforts to address these issues include optimizing organic label design and improving the stability and sensitivity of the detection system.

To characterize the performance of electroactive compounds in biosensing applications, two key factors must be considered. First, it is important to select molecules with redox activities that fall within the potential window in which immuno/biosensors remain reasonably stable. In addition, these compounds should demonstrate a significant ability to conjugate with biomolecules or be efficiently converted into conjugatable forms. A wide variety of redox-active molecules can be used as labels in biosensors, including organic small molecules such as thionine [69,70,71], ferrocene [72,73,74,75,76,77], nile blue [78,79,80], anthraquinone [81,82], MB [83,84,85], gallocyanine [86], natural red [87], indophenol [88], carboxy-X-rhodamine (ROX) [89,90], diamino benzoic acid [91], and organometallic complexes such as pentamethylferrocene [86], [Ru(NH_3_)_6_]^3+^, and [Os(bpy)_2_]^3+^ [92]. Each of these compounds offers distinct redox properties and conjugation capabilities, making them suitable for a variety of biosensing applications. By selecting an appropriate electroactive label and optimizing the sensor architecture, researchers can develop highly sensitive and specific biosensors for a wide range of analytes.

Numerous research investigations have examined the development of labels in biological sensors, with particular emphasis on redox reporters linked to DNA in electrochemical biosensors. Further details on this topic will be discussed in the subsequent sections. In a comprehensive study, Plaxco’s group investigated various types of redox reporters conjugated to DNA in self-assembled electrochemical DNA sensors [86]. According to these studies, the redox reporters thionine, dabcyl, and ROX show no characteristic oxidation–reduction currents. Under basic conditions, free indophenol carboxylic acid is stable and electrochemically active; however, attaching indophenol to DNA proved challenging as it decomposed during conjugation to an amine-terminated DNA in a dimethyl sulfoxide (DMSO) medium. Furthermore, the redox potential of neutral red is approximately −0.7 V, which leads to instability of alkane thiols assembled on gold electrodes. In addition, the manner in which electroactive redox reporters are attached to DNA can influence their redox peak behavior. For example, only a small peak current was observed when the ferrocene reporter was conjugated to the 3’ end of DNA. The stability of these redox-conjugated DNA biosensors is also an essential consideration. The conjugation chemistry, the choice of redox reporter, and the operating conditions of the sensors all play crucial roles in determining the overall effectiveness and reliability of the biosensors.

The findings of Plaxco’s study demonstrate that after 100 cycles of square-wave voltammetric scans in buffer, the methylene-blue-modified DNA biosensor loses only about 20% of its current. In contrast, anthraquinone-, nile-blue-, or 5’-inked ferrocene-based sensors show moderate durability under the same conditions, exhibiting approximately 50% signal loss. Likewise, DNA self-assemblies modified with 3’-linked ferrocene, ferrocene-C5, or pentamethylferrocene display markedly reduced stability after 50 cycles, with a current decrease of roughly 50% of their initial signal. However, the signaling current of anthraquinone-based sensors rapidly decreases during the initial scans before reaching a plateau, whereas the signaling current of gallocyanine-based sensors increases substantially with repeated scanning.

Undoubtedly, degradation of the electroactive redox labels contributes to some of these instabilities. For example, the oxygen-containing environment of aqueous solutions renders the ferrocenium ion unstable, and the scan-induced increase in gallocyanine current is likely caused by redox-driven modification of the reporter. Additionally, part of the degradation can be attributed to the instability of the self-assembled monolayer (SAM) when repeatedly exposed to high or low electrostatic potentials. To address these issues—particularly the tendency of electroactive molecular labels to degrade or bind nonspecifically—researchers have exploited the electrical properties of double-helical DNA. Intercalated redox probe molecules have been employed to monitor and characterize disruptions in DNA base stacking. This strategy leverages the inherent stability and specificity of the DNA double helix, thereby improving the robustness and reliability of DNA-based electrochemical biosensors. Intercalators bind to DNA by inserting a planar aromatic chromophore between adjacent base pairs [93]. Redox-active intercalator-based electrochemical DNA biosensors, in terms of electron transfer through self-assembled DNA monolayers to reporter molecules, can be divided into three models [94], namely:

1. Charge transfer to reporter molecules positioned far from the electrode surface and located above mismatch sites. These molecules are typically situated above regions where mismatches in the DNA sequence occur, enabling the detection of specific mismatches by monitoring changes in the redox activity of the intercalated reporters.
2. Charge transfer to intercalated molecules located before the mismatch and positioned closer to the electrode surface. Electron transfer occurs to these intercalators, and their proximity to the mismatch influences the redox signal. This model helps identify the presence and approximate location of mismatches within the DNA sequence by observing the redox behavior of the intercalated molecules.
3. Direct electron transfer to the electrode surface through pinholes in the monolayer. These pinholes allow direct contact between the electrode and the intercalated redox probes, enabling efficient electron transfer. This model benefits from enhanced electron-transfer rates and is suitable for detecting even minor disruptions in the DNA structure.

Clearly, to distinguish between fully complementary and mismatched targets, the intercalated reporter molecules must be located after the mismatch sites. In contrast, the two other models cannot discriminate between complementary and mismatched targets. This issue becomes more pronounced when attempting to differentiate thermodynamically stable mismatches such as G–A and G–T from complementary strands, as these mismatches remain well-stacked within the duplex [44,95].

Using MB, Barton and colleagues reported that it lacks the sensitivity required to detect the dynamic behavior of a G–A mismatch. To address this issue, the same group performed an electrocatalytic reduction of ferricyanide by MB [96]. In this electrocatalytic process, MB is electrochemically reduced through DNA π-stack-mediated charge transport. The reduced form of MB (leucomethylene blue) is subsequently oxidized back to MB by the ferricyanide oxidizing agent. This cycle generates additional electrons that can flow through MB via DNA-mediated charge transport, thereby enhancing the detection of thermodynamically stable mismatches. However, the penetration of MB below the mismatch may hinder the electrocatalytic effect of ferricyanide due to repulsive interactions between the negatively charged DNA and ferricyanide.

Employing negatively charged intercalators can effectively address this issue. Due to charge repulsion, it is believed that these intercalators penetrate only the very top of the DNA duplex [94,97]. The use of such intercalators has shown that these assays are sufficiently sensitive to detect perturbations in the π-stack, enabling the identification of all possible single-base mismatches, particularly the thermodynamically stable ones, without the need for a catalytic approach. However, despite their advanced detection capabilities and improved detection limits, methods based on redox-active intercalators often suffer from high background signals due to non-specific interactions of redox compounds with the electrode surface or ssDNA. Consequently, any factor that prevents electroactive species in the solution from reaching the electrode surface can hinder accurate identification of the target species, thereby limiting the performance of these methodologies.

As previously mentioned, several factors—such as non-specific adsorption of interfering molecules, steric hindrance, and diffusion-rate limitations—have been identified as contributors to delays in electrochemical response. These delays can lead to false-negative results or inaccurate analytical interpretations. Despite the wide applicability of electrochemical methods, such constraints have hindered the full advancement of personalized healthcare models that rely on these techniques for monitoring and diagnosis. Thus, progress in personalized medicine depends on the development of reliable and efficient electrochemical sensing devices capable of overcoming these obstacles. Due to the aforementioned limitations, it is necessary to employ alternative signal-recording strategies that do not rely on electroactive materials in the measuring medium. Reagent-free and label-free electrochemical approaches are promising candidates for developing versatile biosensors with improved practicality. Using energy-based readout mechanisms as signal-recording agents may be particularly advantageous in this context.

Recording signals without relying on electroactive or enzymatic reactions is possible through the use of the HER, the oxygen reduction reaction (ORR), and water splitting as energy-based signal recorders [35]. These reactions offer high sensitivity, selectivity, and rapid response times, playing crucial roles in various electrochemical processes and attracting significant interest in biosensing applications. HER and ORR, along with water splitting, are fundamental to energy storage and conversion devices such as fuel cells and water electrolyzers, where they convert oxygen and hydrogen ions into water and hydrogen gas, respectively. In the context of biosensing, these reactions provide a distinct signal for detecting specific analytes, including biomolecules and chemical species, by altering the catalytic activity at the electrode–electrolyte interface. This analysis examines the roles of HER, ORR, and water splitting in electrochemical biosensing, exploring the underlying mechanisms and principles that govern these reactions and their interactions with relevant analytes. Integrating HER, ORR, and water splitting into biosensing strategies can enable the development of highly sensitive, fast-response, and cost-effective analytical tools with significant potential for healthcare, environmental monitoring, and other applications [98,99].

Understanding and using the unique characteristics of these reactions will continue to drive advancements in modern analytical research and biosensing technologies, opening new avenues for innovation and application.

Finally, unless otherwise stated, electrode potentials discussed in this review are reported versus the reference electrode employed in the original studies. Where comparison across different systems is required, potentials were converted to the reversible hydrogen electrode scale using standard conversion relationships. Throughout the manuscript, figure captions and data descriptions explicitly specify the reference electrode, electrolyte, and pH.

## 2. Hydrogen Evolution-Based Electrochemical Detection

### 2.1. Principles and Mechanism of HER

**Figure 1:**
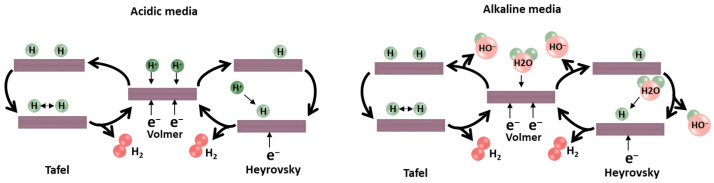
Mechanism HER in acidic and alkaline electrolytes [101].

In general, the reduction of hydrogen and its conversion to hydrogen gas ($H_{2}$) on the electrode surface, in both acidic and alkaline media, is understood through three mechanisms. Initially, the Volmer reaction ((1a) and (1b)) involves the formation of an adsorbed hydrogen atom ($H^{*}$) through the capture of an electron by proton species at the electrode surface. Protons originate from hydronium ions ($H_{3}O^{+}$) in acidic environments and from water molecules in alkaline electrolytes (as illustrated in Figure 1). Subsequently, $H_{2}$ formation occurs either through the Heyrovsky reaction ((1c) and (1d)), the Tafel reaction (1e), or a combination of the two. In the Heyrovsky mechanism, additional protons diffuse from the solution to the electrode, where they react with an electron and $H^{*}$ to produce $H_{2}$. In contrast, the Tafel process involves the recombination of two adjacent $H^{*}$ species to generate $H_{2}$ [100].

Volmer reaction (electrochemical hydrogen adsorption):

(1a) $$\begin{array}{cccc} H_{3}O^{+}+M+e^{-} & ⟷M-H^{+}+H_{2}O\;\;\;\;\;\; & \; & \mathrm{acidic}\;\mathrm{medium} \end{array}$$

(1b) $$\begin{array}{cccc} H_{2}O+M+e^{-} & ⟷M-H^{+}+{\mathrm{OH}}^{-} & \; & \mathrm{alkaline}\;\mathrm{medium} \end{array}$$

Heyrovsky reaction (electrochemical desorption):

(1c) $$\begin{array}{cccc} H^{+}+M-H^{+}+e^{-} & ⟷M+H_{2} & \; & \mathrm{acidic}\;\mathrm{medium} \end{array}$$

(1d) $$\begin{array}{cccc} H_{2}O+M-H^{+}+e^{-} & ⟷H_{2}+{\mathrm{OH}}^{-}+M & \; & \mathrm{alkaline}\;\mathrm{medium} \end{array}$$

Tafel reaction (chemical desorption):

(1e) $$\begin{array}{ccccc} 2M-H^{+} & ⟷M-H^{+}+H_{2}O & \; & \;\; & \mathrm{acidic}\;\mathrm{and}\;\mathrm{alkaline} \end{array}$$

The response mechanism is determined by the slope of the Tafel plot, denoted as *b*. This slope represents the change in potential required for a tenfold increase or decrease in current density. Essentially, *b* serves as an intrinsic characteristic of the catalyst, directly associated with the rate of the HER, as defined by Equation (2). This relationship is established by plotting η against *j*, where *j* denotes the current density and $j_{0}$ represents the exchange current density. A smaller value of *b* indicates faster electron-transfer kinetics, as a smaller increase in potential is needed to achieve a tenfold increase in current density.

(2) $$\begin{array}{c} \eta =b\log\left(j_{0}\right)+b\log\left(j\right) \end{array}$$

The constant *b* can be defined as follows:

- If the Volmer (discharge) step is rapid and the Tafel (chemical desorption) step is rate-determining, b=29mV/dec.
- If the Volmer step is rapid and the Heyrovsky step is rate-determining, b=39mV/dec.
- If the Volmer step itself is rate-determining, b=116mV/dec.

It should be noted that HER-based sensing strategies often operate at potentials close to the hydrogen adsorption/desorption region, where the formation and consumption of adsorbed hydrogen intermediates ($H^{*}$) coincide with significant capacitive contributions at the electrode–electrolyte interface. Under such conditions, faradaic currents associated with the HER may partially overlap with non-faradaic capacitive processes, increasing the risk of signal misinterpretation. Consequently, careful selection of the operating potential, together with appropriate control and blank measurements, is essential to ensure reliable signal attribution in HER-based biosensing. Under standard conditions, the Nernst potential for HER on the normal hydrogen electrode is zero. Nevertheless, to overcome kinetic limitations arising from factors such as high activation energy and low energy efficiency, practical HER requires additional potential. These factors cause an overpotential, defined as $\eta =E-E_{\mathrm{HER}}$, where *E* (or $E_{\mathrm{uncorrected}}$) is the applied potential. In practical systems, internal resistances—such as the resistance of the electrode material, electrolyte resistance, and contact resistances (e.g., wires, equipment)—introduce an additional potential drop. Therefore, an unavoidable series resistance ($R_{s}$) generates an ohmic drop that must be corrected in potential–current density (*E*–*j*) curves. Accordingly, the potential required to drive HER is

$$E=E_{\mathrm{HER}}+\eta +iR_{\mathrm{drop}}$$

where $iR_{\mathrm{drop}}$ is the ohmic drop potential [33]. Typically, two specific values of η are reported:

1. $\eta_{1}$, corresponding to a current density of 1 mA ${\mathrm{cm}}^{-2}$, reflecting the onset overpotential.
2. $\eta_{10}$, corresponding to 10 mA ${\mathrm{cm}}^{-2}$, commonly used to compare electrocatalysts.

Smaller $\eta_{1}$ and $\eta_{10}$ values indicate a lower onset potential and lower overpotential required to reach 10 mA ${\mathrm{cm}}^{-2}$, implying stronger electrocatalytic activity for HER.

### 2.2. Application of HER for Electrochemical Signal Tracing

#### Transition-Metal Dichalcogenides (TMDs)

Small labels with sizes comparable to immune molecules, catalytic activity, and long shelf life are ideal labels for probing biomolecular identification. Exfoliated nano TMDs, with the general chemical formula ${\mathrm{MX}}_{2}$, where M is a transition metal (e.g., W, Mo, Hf) and X is a chalcogen (generally S, Se, or Te), have been used as strong candidates for biomolecule detection due to their size compatibility with biomolecules [102,103,104,105]. In their bulk form, however, TMDs exhibit negligible catalytic activity and therefore must be exfoliated to increase surface area and create catalytically active edge and defect sites. Several liquid-assisted mechanical and chemical exfoliation methods have been reported for reducing the size of TMDs [106,107,108,109,110]. Among these, a solution-dependent bipolar electrochemical exfoliation method based on powder precursors dispersed in aqueous solution has been developed [111,112]. Since the starting material is a powder and no electrode modification is required, this process offers a simple and low-cost exfoliation route.

Bipolar electrochemistry (BE), an electrochemical technique based on the polarization of electrically conducting materials (bipolar electrodes) exposed to an external electric field between two driving electrodes, is used in this process to break down powder precursors into nanosized material. Pumera’s research group has applied highly active TMDs such as ${\mathrm{WS}}_{2}$ [111], ${\mathrm{MoSe}}_{2}$ [112], ${\mathrm{WSe}}_{2}$ [113], and their nanoparticles (NPs) as labels for magneto-immunoassays due to their high catalytic activity toward HER.

**Figure 2:**
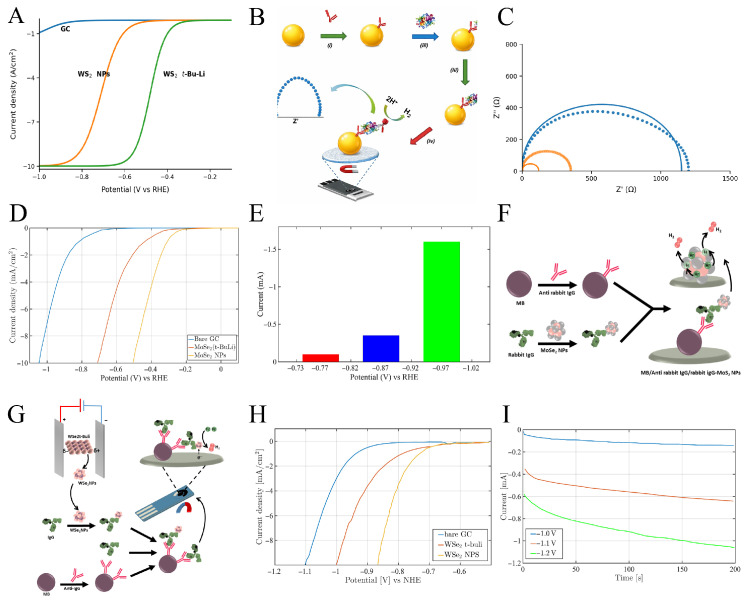
(**A**) LSV of ${\mathrm{WS}}_{2}$ t-BuLi and ${\mathrm{WS}}_{2}$ NPs in $0.5\;MH_{2}{\mathrm{SO}}_{4}$. (**B**) Schematic illustration of magneto-immunoassays for rabbit IgG detection using ${\mathrm{WS}}_{2}$ NPs. (**C**) Nyquist plots of ${\mathrm{WS}}_{2}$ NPs-modified GCE in $0.5\;MH_{2}{\mathrm{SO}}_{4}$ [111]. (**D**) LSV obtained in acidic electrolyte on a different modified glassy carbon electrodes (GCE). (**E**) Chronoamperometry signals recorded at 200s from at ${\mathrm{MoSe}}_{2}$ NPs in $0.5\;MH_{2}{\mathrm{SO}}_{4}$ vs. RHE. (**F**) Magneto-Immunoassay for detection of Rabbit IgG Using ${\mathrm{MoSe}}_{2}$ nano labels via HER Catalysis [112]. (**G**) Bipolar electrochemistry assisted in the synthesis of exfoliated ${\mathrm{WSe}}_{2}$ NPS for Competitive magneto-immunoassay preparation. (**H**) Polarization curve of NP-modified GCE. (**I**) Chronoamperograms of ${\mathrm{WSe}}_{2}$ NPs with different potentials [113]. The figures were adapted from the cited references.

The synthesis of these NPs involved Li intercalation of corresponding TMD sheets using tert-butyl lithium (yielding ${\mathrm{WS}}_{2}$ t-BuLi). By applying a high potential between two platinum driving electrodes and initiating water oxidation at the electrode poles, ${\mathrm{WS}}_{2}$ NPs are fragmented. Linear sweep voltammetry (LSV) measurements of ${\mathrm{WS}}_{2}$ t-BuLi and ${\mathrm{WS}}_{2}$ NPs in $0.5\;MH_{2}{\mathrm{SO}}_{4}$ at a scan rate of 2mV/s revealed that the HER onset potentials for ${\mathrm{WS}}_{2}$ t-BuLi and ${\mathrm{WS}}_{2}$ NPs were −0.502V and −0.66V versus the reversible hydrogen electrode (RHE), respectively (Figure 2A). This shift to more negative overpotential is associated with oxidation of ${\mathrm{WS}}_{2}$ NPs during synthesis.

A rabbit IgG/anti-rabbit IgG–MB complex was formed by binding rabbit IgG to anti-rabbit IgG-modified magnetic beads (MBs). After conjugation of the secondary anti-rabbit IgG with ${\mathrm{WS}}_{2}$ NPs, it recognized rabbit IgG, forming the magneto-sandwich complex anti-rabbit IgG–WS_2_ NPs/rabbit IgG/anti-rabbit IgG–MB, with the final amount of ${\mathrm{WS}}_{2}$ NPs proportional to the rabbit IgG concentration. The magneto-immunosandwich assay for rabbit IgG detection was then performed using ${\mathrm{WS}}_{2}$ nanolabels via HER catalysis and electrochemical impedance spectroscopy (EIS) as the transduction method (Figure 2B). To obtain the EIS polarization potential, Nyquist plots were recorded (Figure 2C) at −0.57V, −0.67V, and −0.77V versus RHE. The semicircle diameter decreased with increasing potential.

Considering the mechanism of HER, its Nyquist equivalent circuit consists of two RC parallel elements in series: one associated with the adsorption step ($R_{\mathrm{ads}}$ and $C_{\mathrm{ads}}$) and the other corresponding to hydrogen evolution to form $H_{2}$ ($R_{\mathrm{ct}}$ and $C_{\mathrm{dl}}$). The calibration curve indicates that the net $R_{\mathrm{ct}}$ decreases at a polarization potential of −0.77V as the protein concentration increases over a broad range between 2ng/mL and 500ng/mL, due to the concentration dependence of the impedimetric signal on the amount of rabbit IgG, with an LOD of 2ng/mL. A relative standard deviation (RSD) of approximately 10% confirms the acceptable reproducibility of the protocol. Subsequently, based on a competitive approach, the same research group employed ${\mathrm{MoSe}}_{2}$ nanolabels for rabbit IgG detection using chronoamperometry [112]. In an acidic electrolyte, the HER catalytic efficiency of ${\mathrm{MoSe}}_{2}$ (t-BuLi) sheets and ${\mathrm{MoSe}}_{2}$ NPs, evaluated via LSV on a GC electrode, revealed overpotentials of −0.524V and −0.722V for ${\mathrm{MoSe}}_{2}$ (t-BuLi) sheets and ${\mathrm{MoSe}}_{2}$ NPs, respectively (Figure 2D).

Although the electrocatalytic activity of ${\mathrm{MoSe}}_{2}$ (t-BuLi) is higher than that of ${\mathrm{MoSe}}_{2}$ NPs, the ${\mathrm{MoSe}}_{2}$ NPs were used as nanolabels for measuring rabbit IgG. This choice is attributed to the size of the ${\mathrm{MoSe}}_{2}$ NPs, which is comparable to that of proteins and smaller than MBs, leading to improved assay reproducibility. To optimize experimental conditions for HER catalysis, the resulting ${\mathrm{MoSe}}_{2}$ NPs were exposed to three different potentials (−0.77V, −0.87V, and −0.97V versus RHE). Figure 2E shows that the current output at 200s is highest when a potential of −0.97V is applied. Furthermore, at this potential, the signal recorded using a bare GCE is negligible.

As shown in Figure 2F, the IgG–MoSe_2_ NPs/anti-IgG–MB complex was prepared by capturing IgG–MoSe_2_ NPs using anti-rabbit IgG conjugated with magnetic beads (MBs), and the concentration of rabbit IgG was determined based on the amount of ${\mathrm{MoSe}}_{2}$ NPs present. Although ${\mathrm{MoSe}}_{2}$ (t-BuLi) sheets show better HER catalytic efficiency, ${\mathrm{MoSe}}_{2}$ NPs were selected as labels because their size is comparable to proteins and smaller than MBs, which improves assay reproducibility. Good selectivity of this magneto-immunoassay was demonstrated by the acceptable linear response from 2 to 500ng/mL, an LOD of 1.23ng/mL, and a RSD of 9.7% across five protein concentrations when a potential of −0.97V was applied in chronoamperometry.

Tafel analysis confirmed that the HER mechanism on ${\mathrm{MoSe}}_{2}$ NPs is based on the Volmer adsorption step. Although this biosensing system is simple and low-cost, the applied potential for HER is still relatively high compared with other reports. As shown in Figure 2G, exfoliated ${\mathrm{WSe}}_{2}$ produced by BE was used to develop a competitive magneto-immunoassay based on HER on an SPE [113]. The electrocatalytic activity of ${\mathrm{WSe}}_{2}$ NPs toward HER in $0.5\;MH_{2}{\mathrm{SO}}_{4}$ showed better performance and a lower onset potential compared with bare GCE and ${\mathrm{WSe}}_{2}$ (t-BuLi) (Figure 2H). This enhanced electrocatalytic activity results from the harsh synthesis conditions during BE (10V), which create new catalytic sites, edges, and defects. Since the slope of the Tafel curve for all three electrodes exceeds 100mV/dec, the rate-limiting step in each case is the Volmer reaction. For IgG detection using chronoamperometry at a constant potential of −1.2V, different concentrations of IgG protein were mixed with MBs/anti-rabbit IgG conjugate and ${\mathrm{IgG/WSe}}_{2}$ and subsequently drop-cast onto the SPE. This potential was chosen because the highest absolute current was observed for the ${\mathrm{WSe}}_{2}$ NP-modified electrode (Figure 2I). Preliminary studies show that a high current is obtained in chronoamperometry (−1.2V, 200s) even in the presence of only IgG protein, indicating high selectivity of the magneto-immunoassay. The amperometric calibration curve, obtained by measuring the current response after 200s, showed a wide linear range from 5 to 500ng/mL for label-free IgG with a low LOD of 100ng/mL.

### 2.3. Black Phosphorus Nanoparticles (BP NPs)

Competitive magneto-immunoassay was used for the detection of free rabbit IgG based on the electrocatalysis of impacting black phosphorus nanoparticles (BP NPs) via HER (proton reduction) [114]. A single-step BE process was performed to synthesize BP NPs by exfoliating and downsizing layered black phosphorus microparticles in a solution containing BP crystals and ${\mathrm{Na}}_{2}{\mathrm{SO}}_{4}$, using two platinum electrodes spaced two centimeters apart with a potential difference of 10V. The higher electrocatalytic activity of BP NPs toward HER was confirmed using LSV and EIS measurements in $0.5\;MH_{2}{\mathrm{SO}}_{4}$. The lower overpotential ($\eta_{10}$) and the dramatic decrease in charge-transfer resistance ($R_{\mathrm{ct}}$) observed for BP NPs ($\eta_{10}=-0.81\;V$, $R_{\mathrm{ct}}$ = 1.39 kΩ), compared with BP macroparticles ($\eta_{10}=-1.24\;V$, $R_{\mathrm{ct}}$ = 37.6 kΩ) and bare glassy carbon ($\eta_{10}=-0.97\;V$), confirm that exfoliation produces more catalytically active edge sites and enhances HER performance.

**Figure 3:**
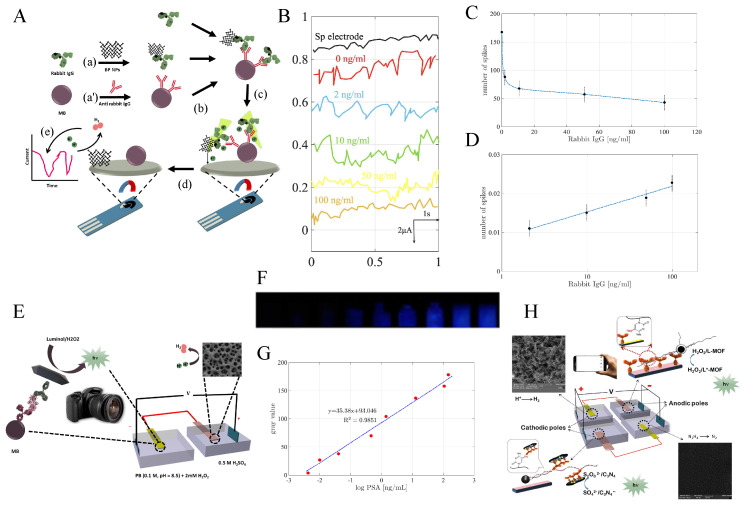
(**A**) Schematic of HER electrocatalysis using BP NPs for the competitive magneto-immunoassay. (**B**) Chronoamperograms of the immunoassay at different rabbit IgG concentrations. (**C**) Spike-count curve and (**D**) Inverse spike counts as a function of rabbit IgG concentration [114]. (**E**) HER-mediated visual ECL detection of PSA. (**F**) Captured images at different PSA concentrations and corresponding signals. (**G**) Calibration curve [120]. (**H**) Visual simultaneous detection of RASSF1A and SLC5A8 tumor suppressor gene methylation [121]. The figures were adapted from the cited references.

As illustrated in Figure 3A, to detect IgG using BP NPs as labels, anti-rabbit IgG was first coupled to tosyl-activated paramagnetic beads (a). A competitive magneto-immunoassay was then designed by mixing rabbit IgG-modified BP NPs (a’) with anti-rabbit IgG-conjugated MBs in the presence of a desired concentration of free rabbit IgG (b). The resulting immune complex was subsequently drop-cast onto a screen-printed electrode in a $1\;M\;H_{2}{\mathrm{SO}}_{4}$ solution (c). The acid induces denaturation of the immune complex, leading to the release of BP NPs, which migrate to and interact with the electrode surface (d). Finally, detection of the IgG concentration was performed via the nanoimpact [115,116,117,118,119] of the HER electrocatalytic behavior of the released BP NPs (e). The acidic medium serves not only as a supporting electrolyte but also as a denaturing agent that facilitates the liberation of BP NPs.

The acidic solution serves not only as a supporting electrolyte but also as a denaturing agent that facilitates the release of BP NPs. The number of spikes (i.e., the impacts generated by BP NPs on the electrode surface) is proportional to the number of nanoparticles attached to the protein, which in turn corresponds to the amount of rabbit IgG. As the concentration of free rabbit IgG increases, the number of spikes decreases due to competition between free antigens and IgG–BP NP conjugates for binding to the anti-rabbit IgG-conjugated MBs, based on the high affinity of the antigen–antibody interaction. At all tested concentrations of free rabbit IgG, the number of spikes generated by the magneto-immunoassay complex is greater than that of the blank control (Figure 3B, black line). The calibration curve obtained using chronoamperometry at an applied potential of −0.88V, based on the spike count within the first 40 s (Figure 3C,D), shows a linear range of 2 to 100ng/mL with an LOD of 0.98ng/mL.

#### 2.3.1. Ruthenium Nanoparticles (Ru NPs)

One effective strategy for achieving high sensitivity in bipolar electrochemistry (BE) is to reduce the potential difference between the anodic and cathodic poles. In this regard, various electrocatalysts in the cathodic compartment have been employed to catalyze the electroreduction of oxygen [122,123], thionine [124], and protons [120,121]. Among these systems, proton reduction using noble-metal electrocatalysts occurs at more positive potentials than the others, which significantly reduces $\Delta E_{\mathrm{elec}}$ and enhances sensitivity. To this end, our group developed a sensitive visual smartphone-based electrochemiluminescence (ECL) method for PSA detection [120] and the simultaneous detection of RASSF1A and SLC5A8 tumor suppressor gene methylation in plasma from thyroid cancer patients [121]. In this approach, ultrasensitive PSA detection was achieved using ECL derived from a high loading amount of luminol encapsulated in MIL-53(Fe)${\mathrm{-NH}}_{2}$ (L@MIL-53(Fe)${\mathrm{-NH}}_{2}$) acting as an accelerator at the anodic pole through a sandwich immunoassay between ${\mathrm{Ab}}_{2}$/L@MIL-53(Fe) and ${\mathrm{MNP/Ab}}_{1}$ [120]. Alternatively, ruthenium nanoparticles electrodeposited on nitrogen-doped graphene-coated Cu foam (fCu/N-GN/Ru NPs) were used to decrease the HER overpotential at the cathodic pole (Figure 3E).

The electrocatalytic activity of fCu/N-GN/Ru NPs toward HER at each modification step was assessed using LSV in $0.5\;M\;H_{2}{\mathrm{SO}}_{4}$ at room temperature with a potential scan rate of 0.5mV/s. Compared with fCu (−377mV), fCu/N-GN exhibits higher electrocatalytic activity, with an overpotential of −213mV at $-10\;\mathrm{mA}\;{\mathrm{cm}}^{-2}$, due to the reduced resistance and enhanced metallic character arising from the injection of *p* electrons from pyridinic nitrogen into the graphene π system. Moreover, the nanostructure of Ru NPs provides additional catalytic benefits. The most impressive HER activity is observed for fCu/N-GN/Ru NPs, which display an overpotential of −114mV—corresponding to reductions of 263mV, 99mV, and 76mV relative to fCu, fCu/N-GN, and fCu/Ru NPs, respectively. In addition, a much smaller Tafel slope was obtained for fCu/N-GN/Ru NPs ($81\;\mathrm{mV}\;{\mathrm{dec}}^{-1}$) compared with fCu ($164\;\mathrm{mV}\;{\mathrm{dec}}^{-1}$), fCu/N-GN ($130\;\mathrm{mV}\;{\mathrm{dec}}^{-1}$), and fCu/Ru NPs ($83\;\mathrm{mV}\;{\mathrm{dec}}^{-1}$), indicating facilitated mass transport between the catalyst and electrolyte as well as faster electron-transfer kinetics at increasing overpotentials. To evaluate the electrochemical behavior of fCu/N-GN/Ru NPs for enhancing the ECL signal at the anodic pole by lowering the voltage required to drive the reactions (i.e., reducing $\Delta E_{\mathrm{elec}}$), the ECL intensity of the ${\mathrm{luminol/H}}_{2}O_{2}$ system was recorded using a PMT detector in the absence and presence of fCu/N-GN/Ru NPs. In the absence of the electrocatalyst, where a Cu sheet served as the cathodic pole of the BPE, luminol emission produced only a weak ECL signal. In contrast, a markedly enhanced ECL intensity—approximately 3.7-fold higher—was observed in the presence of fCu/N-GN/Ru NPs, confirming that the fCu/N-GN/Ru NPs electrode can effectively catalyze HER, facilitate electron transfer through the BPE, and enable sensitive ECL detection.

Due to the urgent demand for portable point-of-care testing, the luminol emission from the MNP/Ab_1_–PSA–Ab_2_/L@MIL-53(Fe)-modified anodic pole of the BPE was captured using a smartphone (Figure 3F). Figure 3G demonstrates a linear correlation between the ECL gray value and PSA concentration, with a detection limit of 0.1pg/mL. Furthermore, our group introduced a novel method for the simultaneous detection of 5-methylcytosine (5mC) in the tumor suppressor genes RASSF1A and SLC5A8 in papillary thyroid cancer using an integrated BE–ECL platform, where the emitted light was recorded by a smartphone camera. In this system, ${\mathrm{Fe}}_{3}O_{4}$@UiO-66-functionalized luminol was placed on the anodic pole of the BPE, and gold nanorods loaded on graphite-like carbon nitride nanosheets (${\mathrm{AuNRs@C}}_{3}N_{4}$ NS) were placed on the cathodic pole (Figure 3H) [121]. As a result of HER at the fCu/N-GN/Ru NPs cathode and electrooxidation of hydrazine at the PC-rGO/PGE anode, this approach not only maintains the same potential difference between poles but also enhances sensitivity by decreasing the $\Delta E_{\mathrm{elec}}$ potential difference.

#### 2.3.2. Platinum Nanoparticles (Pt NPs)

**Figure 4:**
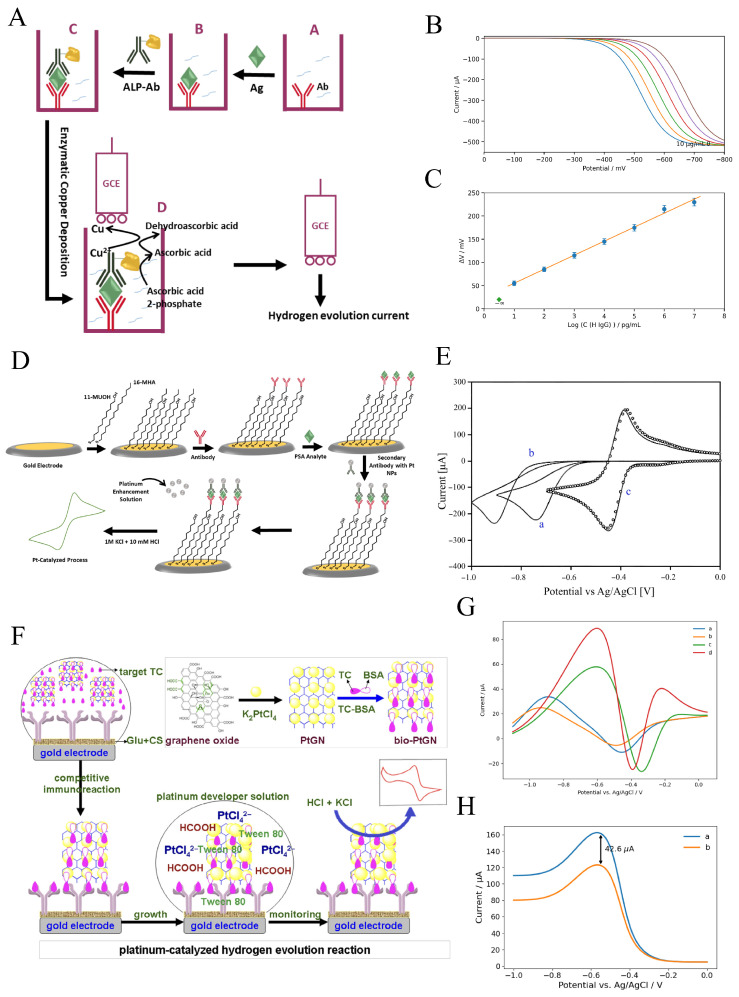
(**A**) Schematic inhibition of HER using the enzymatic Cu deposition on Pt NP-modified GCE. (**B**) LSV response of the immunosensor vs. concentration of hIgG and (**C**) related calibration curve [125]. (**D**) Amplified detection of the PSA based on Pt-catalyzed HER. (**E**) CV of the proton redox; bare Au electrode (a), thiol-modified Au electrode (b), bare Pt electrode (c), and immunosensor in the presence of 10nM PSA [130]. (**F**) Pt-catalyzed HER with Pt-mediated seed growth for detection of TC. (**G**) CV of (a) TC/Glu/CS-modified Au, modified Au in the presence of (b) 50ng/mL TC, and with (c) excess bioPtGN, (d) electrode ‘c’ after incubation with Pt developer solution. (**H**) LSV responses of the electrochemical immunosensors toward (a) zero analyte and (b) 1.0ng/mL TC standards using various labeling probes bio PtGN [131]. The figures were adapted from the cited references.

Electrochemical immunosensors generally use either enzymatic or nanomaterial-based tags to record the signal generated by the analyte. Enzyme-linked electrochemical immunoassays (ELEIA) have shown substantial potential for detecting molecular targets in clinical diagnostics, food analysis, and other fields. In general, the response of ELEIA is measured either through the amperometric detection of electroactive species or through potentiometric monitoring of ionic products generated by enzymatic reactions. However, due to the inherent limitations of amperometric and potentiometric methods in achieving highly sensitive detection, the development of more sensitive strategies remains a major challenge. Signal amplification based on the catalytic capability of Pt to produce strong electrocatalytic currents for the HER offers a promising new approach for enhancing biosensor sensitivity. Since Pt NPs are well known as efficient electrocatalysts for HER, the presence of additional heavy metals can poison their catalytic activity. In this regard, in 2008, Huang and co-workers [125] reported a sensitive method for detecting human IgG (hIgG) based on HER inhibition through enzymatic Cu deposition on Pt NP-modified GCE in an alkaline medium containing ascorbic acid 2-phosphatase (AAP) and ${\mathrm{Cu}}^{2+}$ ions. In this system, after the target hIgG was sandwiched between the capture antibody immobilized on the microtiter plate wells and the alkaline phosphatase (ALP)-conjugated detection antibody, the enzymatic reaction of ALP generated the reducing agent ascorbic acid in glycine–NaOH buffer (pH = 10). Consequently, the reduction of ${\mathrm{Cu}}^{2+}$ ions in solution ((3a)–(3c)) resulted in the selective deposition of copper onto the Pt NP electrode (Figure 4A).

(3a) $$\begin{array}{cc} \mathrm{ascorbic}\;\mathrm{acid}\;2-\mathrm{phosphate} & \overset{\mathrm{ALP}}{\to }\mathrm{ascorbic}\;\mathrm{acid}+H_{3}{\mathrm{PO}}_{4} \end{array}$$

(3b) $$\begin{array}{cc} \mathrm{ascorbic}\;\mathrm{acid}\;+{\mathrm{Cu}(\mathrm{glycin})}_{2}^{2+} & \to \;\mathrm{dehydroascorbic}\;\mathrm{acid}+{\mathrm{Cu}(\mathrm{glycin})}^{+}+H^{+} \end{array}$$

(3c) $$\begin{array}{cc} \mathrm{ascorbic}\;\mathrm{acid}\;+2{\mathrm{Cu}(\mathrm{glycin})}^{+} & \overset{\;\;\mathrm{Pt}\;\;}{\to }\mathrm{dehydroascorbic}\;\mathrm{acid}+2{\mathrm{Cu}}^{0}+H^{+} \end{array}$$

The reason for using GL–NaOH buffer is that ${\mathrm{Cu}}^{2+}$ ions do not precipitate in alkaline media and instead form a stable complex with GL. Owing to the poisoning of the Pt catalyst through hIgG-dependent enzymatic copper deposition, a clear potential shift was observed between the copper-deposited electrode and the undeveloped (copper-free) electrode (Figure 4B). The response of the immunosensor at a constant current of 500 μA showed a linear relationship over the range of 1.0pg/mL to 100pg/mL with a low detection limit of 1.0pg/mL (Figure 4C). In contrast to silver and organic precipitates [126,127,128], copper is chemically and selectively deposited onto Pt NPs, yielding significantly greater sensitivity. Sharma et al. [129] developed a similar strategy for detecting Staphylococcal Enterotoxin B (SEB) using Pt NP-modified GCE based on the HER inhibition approach. Anti-SEB polyclonal antibody-modified SPEs were exposed to various concentrations of SEB toxin to enable selective antigen–antibody binding. After the addition of secondary mouse anti-SEB monoclonal antibodies, the resulting immune complex was incubated with ALP-conjugated anti-mouse antibodies. Finally, the indirect sandwich enzyme-linked immunoassay (ELISA) assembly, together with the Pt NP-modified GCE, was immersed in GL–NaOH buffer (pH = 10) containing the copper deposition solution and AAP.

Under optimized conditions, the enzymatic reaction led to the hydrolysis of ascorbic acid 2-phosphate, which consequently resulted in copper deposition on the Pt NP-modified GCE according to the above mechanism. A negative potential shift in 0.1M HCl was observed for SEB concentrations ranging from 1.0ng/mL to 1.0μg/mL, with a detection limit of 1.0ng/mL. Detection of SEB in buffer samples, without the addition of external tracing labels, provided a simple and reproducible signal. In addition, the ability of Pt NPs to reduce protons and produce high HER currents contributes to increased sensitivity in field analysis. Nanoparticles have also been used as seeds to catalyze further metal precipitation to enhance signal amplification by increasing the amount of deposited material. In most cases, catalytic deposition of Au [132] or Ag [133] on colloidal gold labels has been reported. After dissolution of the deposited metal in acid, highly sensitive biomolecule detection can be achieved using stripping voltammetry. Electrocatalytic reduction of $H^{+}$ using platinum is advantageous for several reasons:

1. Hydrogen undergoes reversible electrochemical reduction on the surface of platinum.
2. Background electroreduction of $H^{+}$ on non-Pt electrodes is negligible because the catalytic activity of platinum is substantially higher than that of other materials.
3. The diffusion coefficient of hydrogen ($1\times 10^{-4}\;{\mathrm{cm}}^{2}\;s^{-1}$) is at least one order of magnitude higher than that of many other redox species.
4. Inorganic acids are highly soluble and stable in aqueous media.

Regarding these benefits, the gap between the electrode surface and the Pt NPs resulting from the presence of antibodies causes slow electron tunneling, which reduces the measurable current. Therefore, a Pt enhancement step based on seed-mediated Pt deposition is necessary. To this end, Zhang et al. [130] proposed a prostate-specific antigen (PSA) immunosensor using Pt seed-mediated growth in a platinum developer solution containing formate, ${\mathrm{PtCl}}_{4}^{2-}$, and Tween 80 (pH 6.5, 1mM) (Figure 4D).

As shown in Figure 4E, the platinum electrode exhibits a lower overpotential for hydrogen reduction, with a formal potential of −0.417V vs. Ag/AgCl, compared with both the bare Au electrode and the thiol-modified Au electrode. The similarity between the voltammogram obtained in the presence of PSA and that of the platinum electrode indicates that the Pt NPs generated by the developer solution were active for the hydrogen evolution reaction. Pt enhancement time is one of the critical factors influencing the electrochemical response of the immunosensor. At short enhancement times (2–5 min), radial diffusion controls mass transport, and a sigmoidal steady-state voltammogram is observed because the spacing between small Pt particles is approximately ten times their diameter, causing each Pt NP to behave as an individual microelectrode. However, when enhancement time exceeds one hour, the Pt NPs completely cover the electrode surface and planar diffusion becomes dominant. The calibration curves show that increasing the enhancement time improves the sensitivity of the PSA immunosensor. The response range improved from 10fg/mL–10pg/mL to 1fg/mL–1pg/mL when Pt enhancement time increased from 10 to 30 min. For further signal amplification, nanoparticles can be coupled with two-dimensional materials. Recent studies show that NP-decorated 2D materials provide promising strategies for biosensing applications. Graphene, as a two-dimensional material, has attracted significant interest owing to its high electron-transfer rate, large surface-to-volume ratio, and excellent electrical conductivity. Que et al. [131] reported the synthesis of platinum/graphene nanosheets (PtGN) via a redox reaction between GO and ${\mathrm{PdCl}}_{4}^{2-}$ and employed them to label tetracycline–bovine serum albumin conjugates (TC–BSA) for the electrochemical determination of tetracycline (TC) as a signal amplification strategy (Figure 4F).

To construct the competitive immunoassay, the anti-TC antibody was first covalently conjugated onto the chitosan-modified Au electrode through glutaraldehyde cross-linking. Subsequently, to determine TC residues on the anti-TC/Glu/CS/Au electrode, the immunosensor was immersed in the platinum developer solution described above. Compared with the anti-TC/Glu/CS/Au electrode in the absence and presence of TC, the TC/anti-TC/Glu/CS/Au in the presence of TC–PtGN exhibited a pair of redox peaks at −0.417 V and −0.384 V in 1.0 M KCl and 10 mM HCl, originating from the Pt NP-based HER. Interestingly, further increases in the redox peak currents were observed after Pt-mediated growth in the platinum developer solution.

Moreover, the advantages of PtGN over the previous immunoassay were demonstrated by employing two labeling strategies: PtGN-labeled TC–BSA and Pt-labeled TC–BSA. The higher peak current of TC–PtGN compared with TC–PtNP can be attributed to the higher loading of Pt NPs and TC–BSA conjugates on the high-surface-area graphene nanosheets (Figure 4G).

When one TC–BSA conjugate binds to anti-TC, additional Pt NPs located on the same graphene nanosheet participate in the HER, resulting in enhanced peak currents (Figure 4H). The sensitivity and linear range of the immunosensor in 10mM HCl + 1.0M KCl, based on the electrocatalytic properties of TC–PtGN toward platinum-catalyzed HER, showed a useful analytical range from 0.05ng/mL to 100ng/mL with a low detection limit of 6pg/mL. Although this method allows determination of various antibiotics by adjusting the target antibodies without relying on enzyme substrates, performing measurements in aqueous environments remains challenging because Pt NPs are highly active in water and are readily influenced by external conditions.

A sensitive label-free electrochemical detection strategy for PSA using electrocatalytic Pt NPs conjugated to a recombinant scFv antibody was reported by Spain et al. [134]. While the bare gold electrode exhibited oxidation and reduction peaks at +1.3V and 0.87V in $0.01\;MH_{2}{\mathrm{SO}}_{4}$, the L-cysteine-modified electrode showed an increased peak current for gold oxide reduction. The increase in microscopic electrode area exposed after L-cysteine modification ($0.076{\mathrm{cm}}^{2}$), relative to the pristine surface ($0.076{\mathrm{cm}}^{2}$), was attributed to the loss of surface gold atoms induced by cysteine binding, leading to surface roughening. The presence of PSA resulted in a decrease in the gold oxide reduction peak current. Formation of the sandwich complex with scFv-labeled electrocatalytic Pt NPs generated metallic platinum at approximately +0.6V through reduction of platinum oxide, and produced hydrogen adsorption/desorption features at about −0.3V. In addition to the hydrogen adsorption/desorption peaks, the peak associated with platinum oxide at 0.2V increased with increasing PSA concentration.

#### 2.3.3. Gold Nanoparticles (Au NPs)

The gold electrode is generally inert toward redox reactions because of the repulsion between hydrogen and oxygen molecular orbitals and the fully occupied *d*-states of gold. In contrast, Au NPs behave differently owing to the large number of unsaturated atoms at their edge sites. In addition, quantum-size effects arising from the electronic structure of Au NPs, including the presence of *d*-orbital electrons, promote enhanced electrostatic interactions. Altogether, these features give Au NPs significant electrocatalytic properties. Their good biocompatibility and high conductivity also make them attractive for biosensor applications. To investigate the electrocatalytic properties of Au NPs toward HER, different concentrations of 20 nm Au NPs were drop-cast onto screen-printed carbon electrodes (SPCEs) and tested in 1M HCl, where no evidence of Au NP aggregation was observed [135].

**Figure 5:**
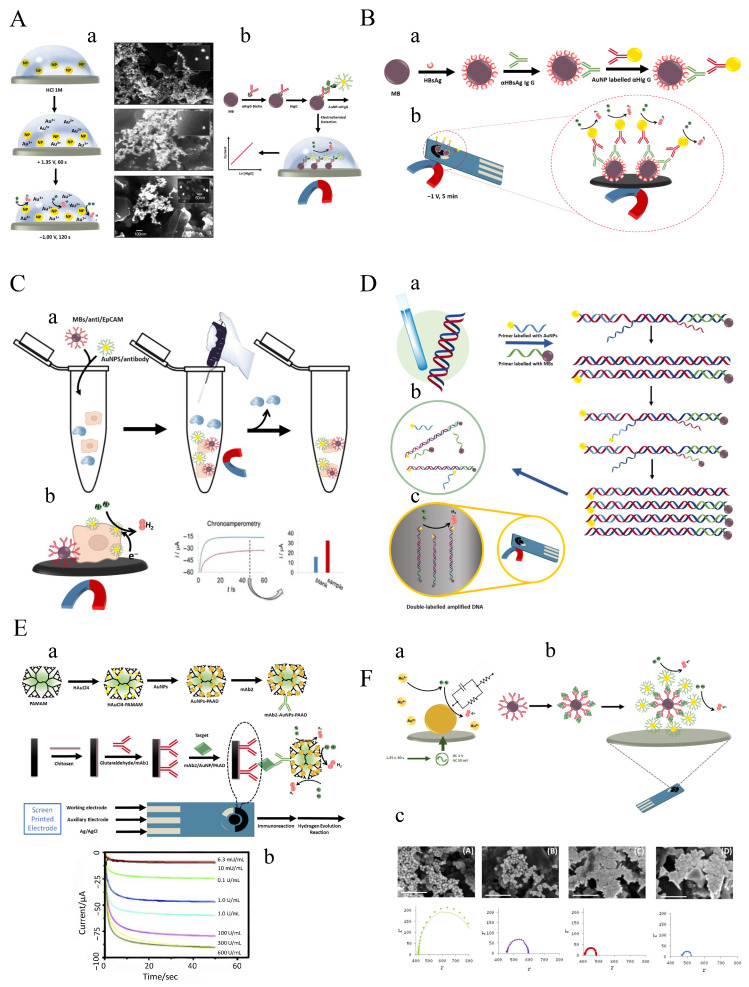
(**A**) (**a**) Schematic illustration of Au NP-catalyzed HER (left) and corresponding SEM images (right); (**b**) magneto-sandwich immunoassay for detection of α-HIgG-B [135]. (**B**) (**a**) Scheme of the sandwich-type immunosensing of human IgG antibodies; (**b**) electrochemical detection based on HER [136]. (**C**) (**a**) Capture of Caco-2 cells by MBs–anti-EpCAM and antibody-conjugated Au NPs in the presence of control cells; (**b**) detection of labeled Caco-2 cells through HER electrocatalysis by the Au NP labels [138]. (**D**) Scheme of the experimental procedure for detection of isothermally amplified DNA using primers labeled with Au NPs and MBs: (**a**) DNA extraction from dog blood; (**b**) isothermal amplification of a kinetoplast-specific region via RPA using dual-labeled primers; (**c**) magnetic capture of the double-labeled amplified product (MB/amplified DNA/Au NP complex) on the reverse side of the SPCE working electrode [145]. (**E**) (**a**) Schematic illustration of CA 19-9 detection via Au NP-stimulated HER; (**b**) chronoamperograms of the developed electrochemical immuno-HER assay at different CA 19-9 concentrations [147]. (**F**) (**a**) Principle of EIS sensing via Au NP-induced HER; (**b**) Au NP-induced HER signal tracer for HIgG detection; (**c**) SEM images and Nyquist plots of Au NPs after potentiostatic cathodic polarization at (**A**) −0.6V, (**B**) −0.8V, (**C**) −1.0V, and (**D**) −1.2V [148]. The figures were adapted from the cited references.

The results showed that with increasing nanoparticle concentration, the HER overpotential shifted to more positive values, and the cathodic current at −1V increased. Studies also demonstrated that pre-oxidation of Au NPs at +1.35V enhances their electrocatalytic activity toward HER. This enhancement is attributed to oxidation of surface gold atoms to Au(III) species. The coexistence of these Au(III) ions with Au NPs promotes an increase in HER electrocatalysis. SEM images collected before and after the pre-oxidation step confirmed the presence of Au NPs (Figure 5A(a)). As shown in Figure 5A(b), the electrocatalytic properties of Au NPs were used for the determination of human IgG (HIgG) based on the HER.

Streptavidin-modified MBs were used to immobilize biotinylated anti-human IgG (HIgG-B). After capture of HIgG from the sample, secondary antibodies conjugated with Au NPs were added to form the sandwich complex (Au NP–HIgG). The relationship between the HER signal recorded by chronoamperometry at −1V and HIgG concentration exhibited a dynamic range of 5ng/mL to 1000ng/mL with a detection limit of 1.45ng/mL. In a similar sandwich-type design, anti-hepatitis B surface antigen (HBsAg) was sandwiched between tosyl-activated magnetic bead platforms and electroactive Au NP labels. Electrochemical detection was then achieved by exploiting the catalytic hydrogen evolution properties of Au NPs in acidic media without prior dissolution of the nanoparticles (Figure 5B(a)) [136].

The HBsAg-immobilized MBs (MB/HBsAg) were exposed to HBsAg IgG antibodies present in serum samples, resulting in the formation of the MB/HBsAg–HBsAg IgG complex. Subsequently, HBsAg IgG antibodies were preferentially captured by Au NP-conjugated goat polyclonal anti-human IgG (HIgG) antibodies, where the amount of HBsAg IgG was directly correlated with the quantity of Au NPs. A catalytic current was then generated by proton reduction, dependent on the amount of α-HBsAg IgG, by maintaining the electrode at +1.35V followed by applying −1.0V (Figure 5B(b)). Muiz et al. [137] investigated and optimized a chronoamperometric Au NP measurement approach. At an applied potential of −1.0V, proton reduction occurs preferentially only in the presence of Au NPs; therefore, the cathodic current recorded at a fixed time is proportional to the number of Au NPs. This strategy was successfully applied for detecting cancer cells cultured on SPEs using specific antibodies tagged with Au NPs, enabling detection of 4000 tumor cells in 700 ^−^L of suspension. A similar approach was later used to identify circulating tumor cells (CTCs) [138].

CTCs are circulating blood cells originating from primary tumors or metastatic tissues. CTC quantification is crucial for monitoring cancer progression, prognosis, and therapeutic response [139,140]. Specific membrane proteins overexpressed on cancer cells are widely exploited as recognition targets in CTC biosensing [141]. The CellSearch^®^ system is the only FDA-approved platform for CTC detection, using ferrofluidic nanoparticles conjugated with epithelial cell adhesion molecule (EpCAM) to enrich tumor cells, followed by fluorescence staining for enumeration [142,143]. However, fluorescence detection is time-consuming, costly, and equipment-dependent. To address these limitations, Costa and colleagues [138] developed an electrochemical detection strategy based on the HER activity of Au NPs, combined with anti-EpCAM-modified MBs as a cell-capture platform. EpCAM is highly expressed in colon adenocarcinoma cells (nearly 100%), making it an ideal biomarker for the Caco-2 cell line. Sandwiching Caco-2 cells between Au NP-labeled primary anti-EpCAM antibodies and secondary anti-EpCAM-modified MBs enabled electrocatalytic hydrogen reduction at −1.0V in 1M HCl by chronoamperometry, allowing quantification of labeled cancer cells (Figure 5C).

Electrochemical analysis of Caco-2 cells showed a linear range of $1\times 10^{3}$ to $3.5\times 10^{4}$ cells with an LOD of $1.6\times 10^{2}$ cells. Using MBs to preconcentrate CTCs on SPEs also enabled detection of approximately $5\times 10^{3}\;{\mathrm{mL}}^{-1}$ cells [144]. Due to the strong catalytic HER activity of Au NP labels, electrochemical detection of isothermally amplified *Leishmania* DNA was demonstrated using primers simultaneously tagged with Au NPs and MBs (Figure 5D) [145]. After amplification, the double-labeled MB/amplified DNA/Au NP complexes were magnetically separated and drop-cast onto SPCEs. Following electrochemical pretreatment at +1.35V, HER currents were recorded at −1.0V, enabling highly sensitive and reproducible detection down to $8\times 10^{-3}$ parasites per amplification reaction and successful discrimination between infected and healthy dog blood samples. A simple and specific immune-sandwich assay based on Au NP-catalyzed HER and superparamagnetic MB platforms was also developed for rapid detection of *Escherichia coli* O157:H7 in meat and water [146].

Chronoamperometric measurements in 1M HCl, after pre-oxidation for 1min at +1.35 V and subsequent application of a negative potential of −1.0 V for 100s, revealed a broad detection range of $10^{2}–10^{5}\;\mathrm{CFU}/\mathrm{mL}$ with LODs of 148, 457, and 309 CFU/mL in buffer solution, minced beef, and tap water samples, respectively. This was achieved using capture MBs conjugated with anti-*E. coli* O157 antibodies (MBs–pECAb) and sandwiching the *E. coli* O157 cells with Au NPs modified with secondary antibodies (Au NPs–sECAb). The same strategy was employed for the detection of Alzheimer’s disease (AD) biomarkers, ApoE and human cerebrospinal fluid (CSF), by using Au NP-based HER as the tracer signal and porous magnetic microspheres (PMM) as the preconcentration platform [149]. The high porosity of PMMs increases the surface area available for antibody immobilization, enhancing the catalytic activity of the captured Au NP electrocatalytic labels and enabling sensitive detection. To achieve high sensitivity in electrochemical immuno-HER assays for low-abundance targets, signal amplification remains a critical requirement.

Due to the limited number of catalytic active sites on individual Au NPs, higher loading of Au NPs in immunosensor construction can significantly enhance electrocatalytic performance by increasing the density of active sites. To this end, Ai-Li Sun [147] employed poly(amidoamine) dendrimer (PAAD) to achieve high-density decoration of Au NPs, leveraging the hundreds of potential conjugation sites available on each dendrimer. Herein, Au NP-catalyzed HER was used to quantify carbohydrate antigen 19-9 (CA 19-9) based on the sandwich immunocomplex formed between ${\mathrm{mAb}}_{1}$-modified SPE and ${\mathrm{mAb}}_{2}$–AuNP–PAAD (Figure 5E(a)). Under optimized conditions—pre-oxidation at +1.35V for 50s followed by holding at −1.0V for 50s in chronoamperometry—the immuno-HER assay exhibited a linear range from 0.01U/mL to 300U/mL with a detection limit of 6.3mU/mL in 2M medium. The 3D Au NP–PAAD nanostructures functioned both as nanocatalysts for HER and as platforms for assembly of the detection antibody (Figure 5E(b)). Importantly, the proposed immuno-HER assay was simple, low-cost, enzyme-free, and required no complex instrumentation, making it a versatile detection methodology. Sensitive detection of IgG based on EIS through HER induced by Au NPs was demonstrated by the Merkoçi group [148] (Figure 5E(a,b)). This EIS–HER approach was developed as an alternative to traditional EIS systems relying on redox couples such as [Fe(CN)_6_]^3−/4−^ or [Ru(NH_3_)_6_]^3+/2+^.

Since the impedance behavior of Au NPs depends on both their concentration and size, initial experiments were performed using 1500pM of 20nm Au NPs in 2M HCl. The electrodes were held at a DC potential of +1.35V for 1min (electro-oxidizing Au to ${\mathrm{Au}}^{3+}$), followed by application of various DC potentials while recording impedance spectra from 0.1Hz to 10kHz. A conventional Randles circuit—including solution resistance ($R_{s}$), double-layer capacitance ($C_{\mathrm{dl}}$), and charge-transfer resistance ($R_{\mathrm{ct}}$)—was used to evaluate HER, since hydrogen adsorption on Au NPs is not apparent.

SEM images of Au NP-modified SPEs after applying different DC potentials showed that Nyquist diagrams obtained at potentials more negative than −1V exhibited increased irregularity and reduced stability (Figure 5E(c)). Conversely, at low AC amplitudes (10mV and 20mV), the impedance response was unstable. Because an AC amplitude of 100mV was too large, an AC perturbation of 50mV was selected as optimal. Under these optimized conditions, Nyquist plots recorded at varying Au NP concentrations demonstrated that $R_{\mathrm{ct}}$ decreased with increasing Au NP concentration. After establishing impedimetric detection of Au NP electrocatalysis and confirming its utility as a transduction method, the system was applied to a conventional immunoassay for protein detection. The Au NP signal was proportional to the number of formed immunoconjugates. The inverse of the measured $R_{\mathrm{ct}}$ yielded a linear biosensing response over a wide dynamic range of 2–500ng/mL IgG, with an LOD of 0.31ng/mL. Finally, Table 1 provides a compact comparison of HER-based electrochemical biosensors, including assay formats, electrolytes, operating conditions, readout modes, and analytical figures of merit, highlighting the versatility of HER as a signal transduction and amplification mechanism.

## 3. Oxygen Reduction-Based Electrochemical Detection

### 3.1. Principles and Mechanism of ORR

The most common element in the earth’s crust is oxygen ($O_{2}$). The oxygen reduction reaction (ORR) is a key process in energy conversion devices, such as fuel cells, and in biological respiration. ORR proceeds via two main pathways: a two-electron mechanism, which converts oxygen to hydrogen peroxide, and a four-electron mechanism, which reduces oxygen directly to water. The corresponding ORR processes are summarized in Table 2 together with their thermodynamic potentials (TP) under standard conditions. The electrochemical reduction of oxygen is a complex multistep process involving several intermediates, and its behavior depends on various factors including the catalyst type, electrode material, and electrolyte composition [52,150].

The use of nanoparticles as labels in HER catalysis typically requires highly acidic conditions, introducing additional steps that increase the overall analysis time. Consequently, employing nanoparticles capable of electrocatalyzing reactions under neutral conditions can overcome this limitation. A new generation of nanoparticles has therefore been developed to catalyze naturally occurring neutral-pH processes such as the water oxidation reaction (WOR) and the ORR.

### 3.2. Application of ORR for Electrochemical Signal Tracing

One of the key goals in developing biosensors is to achieve high sensitivity for detecting low-abundance species. Consequently, signal amplification approaches based on nanoparticles have been widely reported in recent years. Owing to their high surface-to-volume ratio, nanoparticles can load large quantities of enzymes such as horseradish peroxidase, thereby increasing signal intensity and overall sensitivity. However, because oxygen is electrochemically generated during the electrode process, horseradish peroxidase-based electrochemical immunosensors require deoxygenation, which limits their clinical applicability, particularly in point-of-care settings [151,152]. In addition, these systems often require extra reactants to generate a measurable signal, making them less suitable for routine analysis. To address these limitations, gold or silver nanoparticles have been employed as labels, and enzymes such as glucose oxidase have also been proposed. Nonetheless, enzyme-based approaches can be adversely affected by harsh environmental conditions such as extreme pH or elevated temperatures, which may compromise enzyme bioactivity and reduce catalytic performance. The use of Au NPs typically requires chemical oxidation in ${\mathrm{Br}}_{2}$ [47] or electrochemical oxidation in HCl [153], followed by reduction to produce a detectable signal. Moreover, the high potentials needed for Au NP oxidation (around +1.3V) may limit specificity and selectivity. As a result, these methods are difficult to implement for routine or urgent clinical analyses. One strategy to overcome these constraints is to use enzyme-free and label-free systems in electrolytes by employing Pt NPs.

#### 3.2.1. Pt NPs

**Figure 6:**
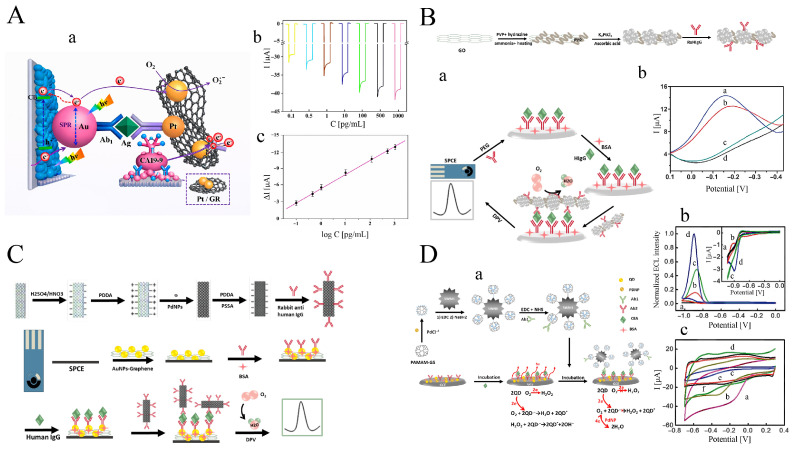
(**A**) (**a**) Photoelectrochemical (PEC) detection of CA19-9 based on Pt/GR-catalyzed ORR as a signal amplifier, (**b**) photocurrent response, and (**c**) corresponding calibration curve [154]. (**B**) (**a**) Signal amplification using PtNDs@GS for sensitive electrochemical immunoassay, (**b**) DPV responses of SPCEs modified with (a) as-prepared PtNDs@GS and (b) PVP-GS in air-saturated PBS, and PtNDs@GS-labeled RaHIgG-bound SPCEs in (c) air- and (d) $N_{2}$-saturated PBS [155]. (**C**) Schematic representation of Pd NPs-decorated CNTs as an efficient ORR signaling tracer for sensitive detection of HIgG. (**D**) (**a**) Electrocatalytic reduction of $O_{2}$ at Pd NPs@PAMAM5/SWNH nanohybrids for CEA detection via an ECL annihilation strategy, (**b**) ECL–potential curves of QD-modified GCE in $N_{2}$-saturated (a), air-saturated (b), $320\;\mathrm{mM}H_{2}O_{2}$ + $N_{2}$-saturated (c), and $O_{2}$-saturated (d) pH 9.0 PBS. Inset: corresponding CVs; (**c**) CVs of Pd NPs@PAMAM5/SWNHs (a), Pd NPs@PAMAM4/SWNHs (b), Pd NPs@PAMAM5 (c), Pd NPs/SWNHs (d), PAMAM5/SWNHs (e), and SWNHs (f) modified GCE in air-saturated pH 9.0 PBS [157]. The figures were adapted from the cited references.

Due to their inherent resistance to interference from reductive species, photocathodic bioassays have shown significant potential for use in real biosample detection. To develop a high-performance photocathodic immunoassay, an enhanced signal amplifier based on a platinum-based nanocatalyst with robust oxygen electroreduction capability was investigated. Typically, a ${\mathrm{CuBi}}_{2}O_{4}$ nanofilm was potentiostatically deposited on an ITO electrode at a constant potential of E=−1.8V vs. ${\mathrm{Hg/Hg}}_{2}{\mathrm{Cl}}_{2}$ in an electrolyte solution containing Cu(NO_3_)_2_ and Bi(NO_3_)_3_ (Figure 6A) [154].

After modification of the nanofilm with Au NPs, the resulting electrode served as the anchoring matrix for capturing the CA19-9 antibody (${\mathrm{Ab}}_{1}$). The signal antibody (${\mathrm{Ab}}_{2}$) was conjugated with Pt NP-decorated graphene oxide nanosheets as the signal amplifier. The Pt/GR nanocatalyst efficiently promoted the electroreduction of oxygen acting as the electron acceptor in the electrolyte when the sandwich immunoreaction occurred, leading to a marked increase in the cathodic photocurrent signal. Using this enhanced signal-amplification strategy, CA19-9 was detected with high sensitivity and specificity using the anti-interference photocathodic immunoassay. As shown in Figure 6B, high loading of Pt NPs onto highly conductive graphene nanosheets (PtNDs@GS) enabled amplified ORR signals for sensitive detection of HIgG in a sandwich-type immunoassay format [155].

The electrocatalytic performance of SPCEs modified with PVP–GS (Figure 6B(b), curve a), PtNDs@GS (curve b), and PtNDs@GS-labeled AbHIgG (curve c) was evaluated in air-saturated PBS (pH 7.4) using DPV. The PtNDs@GS electrode displayed the highest current enhancement. Due to the non-conductive nature of the antibody, a slight current decrease was observed for the PtNDs@GS-labeled AbHIgG electrode. In $N_{2}$-saturated PBS, the PtNDs@GS-labeled AbHIgG-modified SPCE (Figure 6B(b), curve d) exhibited negligible electrocatalytic current. These results indicate that the catalytic reduction of dissolved $O_{2}$ to $H_{2}$O via a four-electron pathway on PtNDs is responsible for most of the amperometric response. Using the oxygen-reduction-based signal tracer, HIgG could be quantified without deoxygenation over the range of 1pg/mL to 10ng/mL.

#### 3.2.2. PdNPs

Using Pd NP-decorated CNTs as labels for the secondary antibody, a highly sensitive sandwich immunosensor was constructed by immobilizing the capture antibody on Au NP-decorated graphene nanosheet-modified SPEs for HIgG detection, based on an efficient ORR signaling tracer (Figure 6C) [156]. The current generated from the electrochemical reduction of oxygen increased with the number of Pd NPs bound to the immunocomplex, enabling HIgG quantification over the range of 50pg/mL to 10ng/mL with an LOD of 40pg/mL. The most significant advantage of this detection approach is that it neither requires a dedicated signal reporter nor demands deoxygenation of the solution. Because of their exceptional benefits—such as high detection sensitivity, spatial separation of excitation and emission, ease of controllability, low background intensity, and the absence of a need for an external excitation light source—ECL sensors have attracted significant interest among electrochemical sensing platforms.

Co-reactant systems have been extensively investigated due to their high ECL efficiency and ability to operate without potential cycling. However, these systems still have drawbacks, including the need for co-reactant transport to the emitter, short radical lifetimes, and possible interference with analyte stability. A promising strategy to reduce the distance between radicals generated from the co-reactant and the luminophore is to decrease the amount of dissolved oxygen or to embed the co-reactant within the luminophore matrix. Quantum dot-based ECL approaches using cathodic oxygen reduction have been employed to enhance ECL intensity. During cathodic potential scanning, the reduction of dissolved oxygen produces $H_{2}O_{2}$, which reacts with electron-injected quantum dots (QDs) to form excited QDs, thereby generating ECL emission. Denoting the formed radical anions as ${\mathrm{QD}}^{-\circ }$, the quantum dot-based ECL pathway in the presence of $O_{2}$ as the co-reactant is described in Equation (4).

(4a) $$\begin{array}{cc} \mathrm{QD}\;+\;e^{-} & ⟶{\mathrm{QD}}^{-\circ } \end{array}$$

(4b) $$\begin{array}{cc} Q_{2}\;+\;2H_{2}O\;+\;2e^{-} & ⟶H_{2}O_{2}\;+\;2{\mathrm{OH}}^{-} \end{array}$$

(4c) $$\begin{array}{cc} {\mathrm{QD}}^{-\circ }\;+H_{2}O_{2} & ⟶2{\mathrm{OH}}^{-}+2{\mathrm{QD}}^{-\circ } \end{array}$$

(4d) $$\begin{array}{cc} 2{\mathrm{QD}}^{-\circ }\; & ⟶\mathrm{QD}\;+\;h\nu \end{array}$$

Meanwhile, any factor that reduces oxygen to water via a four-electron process will suppress the QD ECL emission (“turn-off” effect). This principle was exploited to develop a signal amplification strategy for CEA detection by reducing the $O_{2}$ co-reactant using highly loaded poly(amidoamine) (PAMAM) dendrimer-encapsulated Pd NPs anchored on single-walled carbon nanohorns (SWNHs or SWCNHs), as shown in Figure 6D(a) [157]. The CdTe QD-modified GCE exhibited strong ECL intensity in air-saturated PBS due to the electroreduction of $O_{2}$ to $H_{2}O_{2}$. As shown in Figure 6D(b), sequential modification of the electrode with ${\mathrm{Ab}}_{1}$, BSA, and 100ng/mL CEA caused a decrease in ECL intensity because the inert protein layers acted as an electron barrier and hindered mass transport of the $O_{2}$ co-reactant. The greatest decrease in ECL intensity was observed when the sandwich-type immunoassay was completed in the presence of ${\mathrm{Ab}}_{2}$/Pd NPs@PAMAM5/SWNH. The significantly enhanced quenching efficiency resulted from the electrocatalytic activity of the Pd NPs, which lowered the concentration of the $O_{2}$ co-reactant by promoting its four-electron reduction to water during ORR, thereby suppressing QD ECL emission.

In order to investigate the effect of different electroreduction pathways of $O_{2}$, various nanomaterial-modified GCEs were examined using CV in air-saturated pH 9.0 PBS. Pd NPs@PAMAM5 (Figure 6D(c), curve d) exhibits a greater ORR current than Pd NPs/SWNHs (Figure 6D(c), curve c), indicating that electron transport is enhanced in the presence of SWNHs due to their high conductivity. In contrast, the absence of ORR current for SWNHs and PAMAM5/SWNHs (Figure 6D(c), curves e and f) suggests that SWNHs alone are unable to reduce oxygen. The highest ORR current is observed for the Pd NPs@PAMAM5/SWNHs-modified GCE (Figure 6D(c)), confirming the synergistic effect between Pd NPs, the dendrimer, and the conductive nanohorn scaffold. Pd NPs@PAMAM5/SWNHs efficiently consume the $O_{2}$ coreactant via ORR in the QD-based ECL system, suppressing the formation of the excited state and thereby quenching the ECL emission. Thus, Pd NPs@PAMAM5/SWNHs can serve as an effective signal-tracing tag in QD-based ECL immunoassays. This immunosensor not only provides a wide linear range (from 100ng/mL to 1pg/mL) and high sensitivity (LOD: 0.47pg/mL) but, importantly, does not require deoxygenation during the assay.

### 3.3. Pt-Based Bimetallic

Although noble metal nanoparticles provide low detection limits with high sensitivity, their high cost and potential activity degradation limit their practical applications. To address these issues, alloying noble metals with 3d-transition metals offers an effective strategy to reduce cost while enhancing catalytic activity and electrical conductivity due to the synergistic effects between the bimetals [158,159,160]. Using the synergistic conductivity and catalytic properties of alloyed Pt–Ag atoms, an ultrasensitive, label-free electrochemical immunosensor based on bimetallic PtAg nanowire-catalyzed oxygen reduction was developed for the detection of alpha-fetoprotein (AFP) [161].

Exposure of chitosan-modified GCE to one-dimensional Pt–Ag nanowires (PtAgNW/CSE) in air-saturated PBS produced enhanced oxygen-reduction peak currents. The electrode behavior showed a decrease in ORR current after conjugation of anti-AFP via physico-electrostatic adsorption onto PtAgNW/CSE, followed by interaction between AFP and anti-AFP. Furthermore, no ORR peak was observed for the AFP/anti-AFP/PtAgNW/CSE electrode in $N_{2}$-saturated PBS, confirming that these nanoparticles can be used as electrochemical tags for AFP detection. The higher current and more positive onset potential of the PtAgNW/CSE-modified GCE, compared to commercial Pt/C in $O_{2}$-saturated $0.5\;MH_{2}{\mathrm{SO}}_{4}$ at a defined rotation rate, can be attributed to the increased number of active Pt sites arising from Ag incorporation and the synergistic Pt–Ag interactions. Based on the Koutecký–Levich equation and corresponding plots at different rotation rates, the number of electrons transferred (*n*) was calculated as 4.01 (approximately 4), indicating that oxygen is reduced to water via a four-electron pathway. A continuous decrease in ORR current with increasing AFP concentration, within the linear range of 0.001ng/mL to 36ng/mL and with a detection limit of 0.2pg/mL, highlights the promise of PtAg nanowires as highly sensitive biomarker detection platforms.

**Figure 7:**
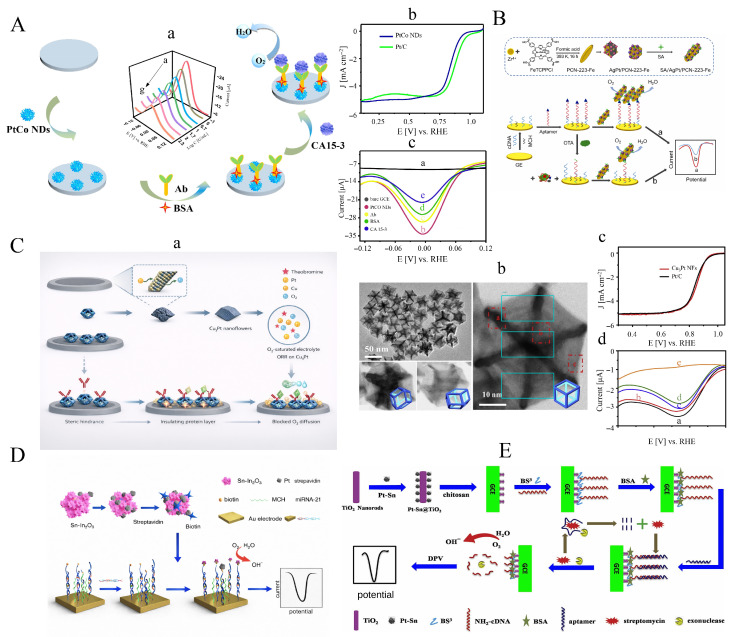
(**A**) (**a**) Detection of CA15-3 based on PtCo NDs-catalyzed $O_{2}$ reduction, (**b**) LSV curves of PtCo NDs and Pt/C in $O_{2}$-saturated 0.1mol/L KOH electrolyte, (**c**) Step-by-step modification of the GCE in air-saturated PBS [162]. (**B**) Electrochemical aptasensing of OTA using the catalytic activity of PtAg/PCN-223-Fe/GCE toward ORR [163]. (**C**) (**a**) Label-free ${\mathrm{Cu}}_{3}$Pt NFs-based immunosensor for AFP quantification, (**b**) EM images of ${\mathrm{Cu}}_{3}$Pt nanoflowers adapted from Ref. [168], showing (i) a low-magnification overview and (ii) a high-resolution TEM image with selected surface regions (Regions I–III) highlighted. Corresponding magnified views illustrate local structural features of the nanoflowers, (**c**) ORR polarization curves of ${\mathrm{Cu}}_{3}$Pt NFs and Pt/C catalysts in $O_{2}$-saturated electrolyte, (**d**) DPV curves of ${\mathrm{Cu}}_{3}$Pt NFs (curve a), ${\mathrm{Ab/Cu}}_{3}$Pt NFs (curve b), ${\mathrm{BSA/Ab/Cu}}_{3}$Pt NFs (curve c), and ${\mathrm{AFP/BSA/Ab/Cu}}_{3}$Pt NFs (curve d) modified electrodes in air-saturated PBS, and ${\mathrm{Cu}}_{3}$Pt NFs-modified electrode (curve e) in $N_{2}$-saturated PBS [168]. (**D**) Design of an efficient Pt/Sn–In_2_O_3_ electrochemical tracer to promote the ORR for miRNA detection [169]. (**E**) Streptomycin aptasensor based on amplified electrocatalytic activity of Pt–Sn@TiO_2_ for ORR [170]. The figures were adapted from the cited references.

Due to the enhanced ORR signals generated by Pt–Co nanodendrites (NDs), a highly sensitive label-free electrochemical CA15-3 assay was developed (Figure 7A(a)) [162]. The ORR performance of PtCo NDs was evaluated using LSV in an $O_{2}$-saturated 0.1mol/L electrolyte (rotation rate: 1600 rpm; scan rate: 5mV/s). As illustrated in Figure 7A(b), the PtCo NDs exhibited a substantially higher onset potential than Pt/C (0.95V versus 0.92V vs. RHE). These results demonstrate the exceptional catalytic activity of PtCo NDs toward the ORR, attributed primarily to their uniform dendritic nanostructures, high specific surface area, and abundance of accessible active sites.

The analytical performance of PtCo NDs for electrocatalytic detection of CA15-3 was assessed through stepwise modification of the GCE (Figure 7A(c)). After immobilization of PtCo NDs (curve b), the peak current at 0.005V increased markedly. Subsequent incubation with the antibody (curve c) and BSA (curve d) caused significant current decreases. A further decline was observed upon binding of CA15-3 (curve e), attributable to the immunocomplex creating substantial hindrance to electron transport. The electrochemical responses obtained with increasing CA15-3 concentration clearly display a “signal-off” behavior (Figure 7A), showing a logarithmic dependence on CA15-3 concentration over the range 0.1–200U/mL, with a low detection limit of 0.0114U/mL. Using AgPt bimetallic nanoparticles decorated on an iron-porphyrinic MOF (PCN-223-Fe), an electrochemical aptasensor for ochratoxin A (OTA) has been developed (Figure 7B) [163]. The electrocatalytic activity of differently modified electrodes toward oxygen reduction was investigated by DPV in pH 6.0 oxygen-saturated PBS. The results demonstrated a pronounced synergistic effect between PCN-223-Fe and AgPt, leading to a substantial enhancement in the catalytic activity of PtAg/PCN-223-Fe/GCE toward the ORR. The biotinylated OTA aptamer-modified GCE captured streptavidin (SA)-modified AgPt/PCN-223-Fe, producing a significant ORR current. Owing to the strong affinity between the aptamer and OTA, formation of the aptamer–OTA complex caused detachment of the AgPt/PCN-223-Fe composite, resulting in a decrease in catalytic current. The proposed method exhibited a wide linear range from 20fg/mL to 2ng/mL and a low detection limit of 14fg/mL.

Beyond compositional tuning, morphological control represents another critical aspect for improving nanocatalysts due to the structure–activity relationship. Metal nanoframes (NFs) have recently gained considerable attention owing to their large surface area, three-dimensional (3D) architecture, and chemical stability [164,165,166,167]. Leveraging the enhanced catalytic currents of the ORR on rhombic dodecahedral ${\mathrm{Cu}}_{3}$Pt NFs, a novel label-free immunosensor was established for the quantification of AFP (Figure 7C(a)) [168]. Here, solvothermally synthesized ${\mathrm{Cu}}_{3}$Pt NFs (Figure 7C(b)) were obtained using theobromine as a structure-directing agent and reductant, together with cetyltrimethylammonium chloride (CTAC) as a co-structure-director. The synthesis involves two main steps:

1. Selective formation of Cu solid nanocrystals with a dodecahedron-like morphology directed by theobromine.
2. Galvanic displacement of surface Cu atoms with platinum salt under high temperature and pressure.

ORR in $O_{2}$-saturated 0.1mol/L KOH solution using linear sweep voltammetry (LSV) shows behavior similar to commercial Pt/C, with two distinct regions: a diffusion-limiting current region below 0.80V and a mixed kinetic–diffusion-controlled region between 1.05V and 0.80V, indicating that ${\mathrm{Cu}}_{3}$Pt NFs exhibit significantly enhanced activity toward the ORR (Figure 7C(c)). The linearity of the Koutecký–Levich plots of ${\mathrm{Cu}}_{3}$Pt NFs at various rotation rates indicates that the total electron transfer number (*n*) is 4, demonstrating that most oxygen molecules are directly reduced to water via a four-electron pathway. Incorporation of Cu into Pt typically increases lattice strain, decreasing the surface affinity for ${\mathrm{OH}}_{\mathrm{ads}}$ and thereby improving ORR efficiency. ORR, a crucial reaction in electrochemical energy systems and an important process in human physiology, provides a promising platform for signal amplification in immunosensing.

As shown in Figure 7C, the ${\mathrm{Cu}}_{3}$Pt NFs/GCE exhibits the highest peak current in air-saturated PBS (pH 7.4) at 0.05V (Figure 7C, curve a). In contrast, the current disappears in $N_{2}$-saturated electrolyte (Figure 7C, curve e), confirming that the peak originates from $O_{2}$ electroreduction. Sequential electrode modification with Ab (Figure 7C, curve b), BSA (Figure 7C, curve c), and AFP (Figure 7C, curve d) results in a progressive decrease in the ORR signal due to steric hindrance and insulating effects from protein layers. The current decreases linearly over the AFP concentration range of $0.1–10^{4}\;\mathrm{pg}/\mathrm{mL}$, with a LOD of 0.033pg/mL, demonstrating that the proposed immunosensor enables ultrasensitive detection of AFP. Because surfactants such as CTAB are difficult to remove completely from nanoparticle surfaces, their use in fabricating architected nanomaterials for enhanced electrocatalytic performance can negatively affect catalytic activity.

Zhang et al. [169] designed an efficient Pt/Sn–In_2_O_3_ electrochemical tracer to promote the ORR for miRNA detection (Figure 7D). Due to strong steric hindrance, the immobilized hairpin structure of the biotinylated capture probe does not interact with streptavidin (SA)-functionalized Pt/Sn–In_2_O_3_. Upon hybridization of the capture probe with the target miRNA, the hairpin structure opens and the biotin group moves away from the electrode surface. Consequently, the modified electrode captures SA-functionalized Pt/Sn–In_2_O_3_, producing a significant ORR signal in $O_{2}$-saturated solution. Using SA/Pt/Sn–In_2_O_3_ as an electrochemical tracer, a miRNA biosensor was developed with a detection limit of 1.92fM and a linear range from 5pM to 0.5fM.

Due to their large surface area, excellent stability, and ability to preserve biomolecular activity, ${\mathrm{TiO}}_{2}$ nanoparticles are increasingly used as scaffolds in biosensors [171,172]. As reported in [171,172], the Pt–Sn@TiO_2_ nanocomposite exhibits enhanced electrocatalytic activity toward the ORR owing to the synergistic interaction between Pt and Sn (Figure 7). The composite was synthesized by decorating ${\mathrm{TiO}}_{2}$ nanorods with Pt and Sn nanoparticles. A detection limit of 20 pM for streptomycin was achieved through exonuclease-assisted target recycling combined with Pt–Sn@TiO_2_-based signal amplification. In this approach, ORR catalysis is suppressed by dsDNA on the Pt–Sn@TiO_2_-modified electrode. In the presence of streptomycin, exonuclease digests the aptamer and the streptomycin–aptamer complex, exposing the Pt–Sn surface and restoring ORR. The use of a bimetallic Pt–Sn alloy resistant to oxidation, together with a ${\mathrm{TiO}}_{2}$ support that provides excellent stability, is a key feature of this sensing platform.

In [173], sodium pyrrolidone-5-carboxylate (PCA–Na), an environmentally benign growth-directing agent, was used to synthesize core–shell Au@Pt nanocrystals (NCs) to enhance electrocatalytic performance by controlling nanoparticle morphology. The electrocatalytic activity of Au@Pt NCs toward the ORR, comparable to that of commercial Pt/C, makes them strong candidates for signal amplification in electrochemical biosensors. A prostate-specific antigen (PSA) immunosensor was fabricated by immobilizing PSA antibodies on Au@Pt NC-modified GCE. The ORR current decreased markedly following the specific antigen–antibody interaction. The immunocomplex hindered access of the redox probe in $O_{2}$-saturated buffer (0.1 M PBS containing 0.15 M NaCl, pH 7.4) to the electrode surface, enabling ultrasensitive PSA detection.

### 3.4. Au-Based Bimetallic

**Figure 8:**
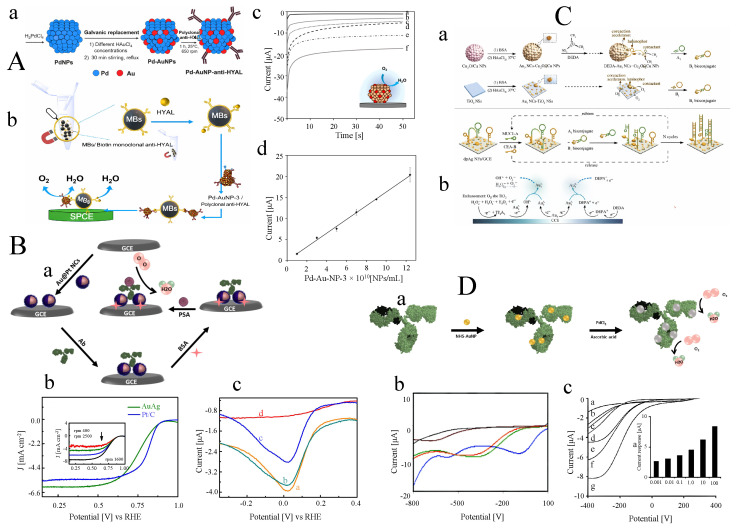
(**A**) (**a**) Synthesis route of polyclonal anti-hyaluronidase (HYAL)-modified Pd–Au NPs, (**b**) Signal amplification for sensitive detection of HYAL using Pd–Au NP-3 tags, (**c**) Chronoamperograms recorded at −0.45V for 50 s at different Pd–Au NP-3 concentrations, (**d**) Recorded currents at 5s versus Pd–Au NP-3 concentration [174]. (**B**) (**a**) AuAg HNCs-catalyzed ORR for construction of the CA19-9 immunosensor, (**b**) Comparison of ORR polarization curves of AuAg HNCs and Pt/C catalysts in $O_{2}$, (**c**) DPV curves of AuAg HNCs (curve a), ${\mathrm{Ab}}_{1}$/AuAg HNCs (curve b), and ${\mathrm{Ab}}_{1}$/CA19-9/AuAg HNCs (curve c) modified electrodes in air-saturated PBS, and AuAg HNCs-modified electrode (curve d) in $N_{2}$-saturated PBS [175]. (**C**) (**a**,**b**) Schematic diagram showing fabrication of the ECL aptasensor [176]. (**D**) (**a**) Preparation of Au/Pd-modified antibodies, (**b**) LSV of GCE modified with NP–antibody at 0, 15, 30, and 60 min Pd deposition (brown, blue, green, and orange lines, respectively) and at 15 min deposition after degassing (black line), (**c**) LSV response with increasing concentrations of TNF-α [177]. The figures were adapted from the cited references.

Toyos-Rodríguez et al. [174] reported that bimetallic Pd–Au NPs with an optimal Pd–Au ratio exhibit outstanding electroactivity toward the ORR. Pd NPs were first synthesized in the presence of polyvinylpyrrolidone using an alcohol reducing agent, followed by selective incorporation of Au atoms through a galvanic replacement reaction (Figure 8A(a)). The effect of Au incorporation into Pd nanoclusters on ORR performance was initially evaluated using linear sweep voltammetry (LSV) on SPCEs in 10 mM PBS (pH 7.4). The results showed that the highest oxygen-reduction activity occurs when the Au content reaches approximately 30%. Notably, the onset potential of the Pd–Au NP-3 (30%) modified SPCE shifts to −0.18V, accompanied by a substantially increased current. Using magnetic beads as platforms, these optimized Pd–Au NPs were subsequently employed as electrochemical labels for hyaluronidase wound infection detection (Figure 8A(b)). At an applied potential of 0.45 V, the amperometric response recorded at 5 s increases with Pd–Au NP-3 concentration due to their catalytic activity toward the ORR (Figure 8A(c,d)). The electrochemical signal increased linearly with hyaluronidase concentration from 125ng/mL to 4600ng/mL, yielding a good linear correlation and a detection limit of 50 ng/mL.

In 2017, Wang et al. [175] introduced hollow-architecture AuAg nanocrystals (AuAg HNCs) prepared using ascorbic acid and hydrazine as reducing agents, together with polycytidylic acid (PCA) as a green growth-directing agent (Figure 8B(a)). Owing to its amine and phosphate functional groups, PCA provides strong electrostatic and chelating interactions with metallic precursors, enabling effective control over nanostructure size and morphology. Compared with Pt/C, the ORR polarization curve of AuAg HNCs exhibits markedly enhanced electrocatalytic activity—showing higher current density and more positive onset potential—in $O_{2}$-saturated 0.1 mol/L KOH (Figure 8B(b)). This enhanced activity is attributed to the unique hollow morphology, which provides abundant active sites, as well as synergistic effects between Au and Ag.

To investigate the number of electrons involved, the ORR was recorded at different rotation rates. Based on the Koutecky–Levich (K–L) equation and the corresponding plots of current density versus the square root of the rotation rate, the number of transferred electrons was calculated to be approximately 4, demonstrating that $O_{2}$ is directly reduced to $H_{2}$O via a four-electron pathway. Owing to the high electrocatalytic activity of AuAg NCs toward ORR, a signal-off, label-free electrochemical CA19-9 immunosensor was proposed. To this end, the performance of the AuAg NCs-modified electrode in PBS (pH 7.4) was evaluated. A sharp reduction peak in the air-saturated electrolyte (Figure 8B, curve a), in contrast to the negligible peak in $N_{2}$-saturated PBS (Figure 8B, curve d), together with the decreased peak current after ${\mathrm{Ab}}_{1}$ immobilization (Figure 8B, curve b) and subsequent CA19-9 binding, clearly indicates that the proposed approach enables CA19-9 detection using $O_{2}$-reduction as the signal tracer. The improved analytical performance—reflected in a low LOD of 0.23U/mL and a wide linear range of 1–30U/mL—is comparable to or even superior to previous reports.

Zhou et al. [176] employed serum–albumin-stabilized ${\mathrm{Au}}_{25}$ nanoclusters (${\mathrm{Au}}_{25}$ NCs) as bipolar ECL probes for the simultaneous detection of CEA and MUC1 (Figure 8C). In this strategy, ${\mathrm{TiO}}_{2}$ nanosheets served as a cathodic coreactant accelerator by catalyzing the reduction of $O_{2}$, thereby enhancing cathodic ECL emission. Conversely, ${\mathrm{Cu}}_{2}$O@Cu functioned as an anodic coreactant accelerator, boosting anodic ECL emission by promoting the oxidation of N,N-diethylethylenediamine (DEDA). This dual-accelerator design avoids cross-reactions between luminophores and enables simultaneous detection of both biomarkers. As a result, CEA and MUC1 were detected with LODs of 0.43pg/mL and 5.8fg/mL, respectively.

Seed-mediated growth was employed to prepare Au/Pd core–shell nanoparticles in the presence of antibody. To obtain 1.4nm Au NPs-modified anti-tumor necrosis factor (TNF-α) antibody, sulfo-NHS-activated Au NPs were reacted with the antibody (Figure 8D(a)) [177]. For this strategy, each NP must act as a single functional unit to avoid antibody cross-linking and aggregation. Subsequently, Au/Pd core–shell NPs were synthesized by depositing ${\mathrm{PdCl}}_{2}$ onto the Au NP-modified antibody using ascorbic acid as the reducing agent. As shown in Figure 8D(b), different Pd deposition times yield distinct electrocatalytic behaviors toward the ORR. As expected, the Au NP/Ab-modified GCE exhibits only a small oxygen reduction wave at approximately −650 mV (Figure 8D(b), brown) in 0.1 M PBS (pH 7.4). After 15 min of Pd deposition, the Au NP diameter increases to roughly 2–2.5 nm due to ${\mathrm{Pd}}^{2+}$ reduction, resulting in two reduction peaks at −160 mV and −570 mV corresponding to the two-electron reduction of oxygen to $H_{2}O_{2}$ and subsequent reduction to $H_{2}$O, respectively (Figure 8D(b), blue). With longer deposition times, the nanoparticle diameter increases to approximately 2–8 nm, producing a broad oxygen reduction peak near −400 mV (Figure 8D(b), green and orange). The lack of further changes at prolonged deposition (Figure 8D(b), orange, 30 min) indicates full coverage of the Au core and behavior characteristic of bulk Pd. The lower ORR potential of the electrocatalytic antibody makes it more suitable for sensing applications than a non-catalytic antibody (Figure 8D(b), brown).

In conventional sandwich immunosensors, efficient electron transfer requires that the electrocatalytic NP-modified detection antibodies remain sufficiently close to the electrode surface. However, as reported in [177], immobilization of primary antibodies via EDC/NHS coupling between carboxyl groups of electropolymerized aminobenzoic acid and the amine groups of capture antibodies showed no significant response change, even at a saturated TNF-α concentration (500ppb). Alternatively, immobilizing diazonium-modified antibodies through electrodeposition produced a porous film with unique pinhole sites ideally suited for immunosensing. Examination of sandwich immunoassays using diazonium-based capture antibody immobilization revealed that increasing TNF-α concentration (from 1ppt to 100ppb) led to higher ORR peak currents due to the accumulation of Au/Pd core–shell NPs. Using electrocatalyzed ORR as the readout not only eliminates the need for external reagents but also simplifies sensor fabrication (Figure 8D(c)).

### 3.5. QDs-Based Detection

**Figure 9:**
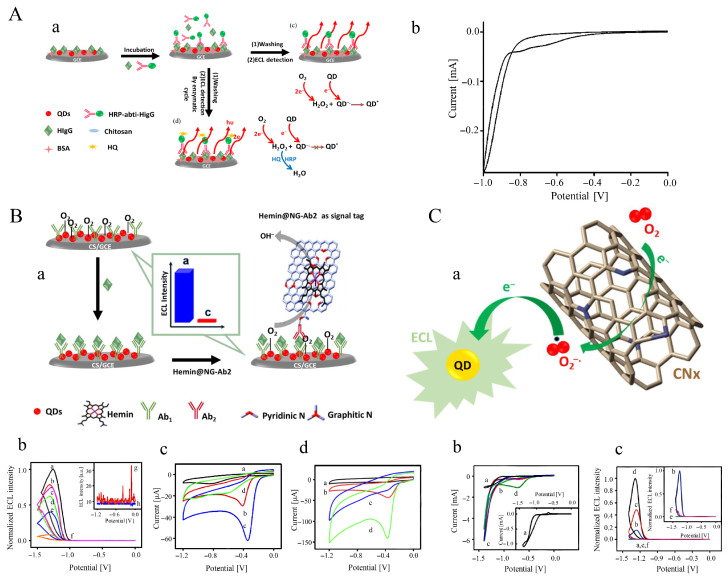
(**A**) (**a**) Enzymatic amplification through consumption of $H_{2}O_{2}$. (**b**) CV curve of GCE/QDs/chitosan–HIgG/HRP–anti-HIgG in air-saturated buffer [178]. (**B**) (**a**) QD-based ECL immunoassay using hemin@NG as a signal tracer. (**b**) ECL responses of the immunosensor in air-saturated 0.1M pH 8.0 PBS: (a) before and after incubation with 10ng/mL CEA, followed by modification with (b) ${\mathrm{Ab}}_{2}$, (c) NG-labeled ${\mathrm{Ab}}_{2}$, (d) HRP-labeled ${\mathrm{Ab}}_{2}$, (e) hemin@GO-labeled ${\mathrm{Ab}}_{2}$, and (f) hemin@NG-labeled ${\mathrm{Ab}}_{2}$. Inset: ECL behaviors of (g) GO- and (h) NG-modified GCEs. (**c**) CVs of the immunosensor in air-saturated 0.1M pH 8.0 PBS after incubation with 10ng/mL CEA and subsequent modification with (a) HRP-, (b) hemin@GO-, (c) hemin@NG-, and (d) NG-labeled ${\mathrm{Ab}}_{2}$. (**d**) CVs of (a,b) hemin- and (c,d) hemin@NG-modified GCEs in (a,c) $N_{2}$-saturated and (b,d) air-saturated 0.1M pH 8.0 PBS [179]. (**C**) (**a**) NCNT-catalyzed reduction of $O_{2}$ for enhanced ECL emission. (**b**) CVs and (**c**) ECL curves of CdS QDs-modified GCE in (a) $N_{2}$-saturated, (b) air-saturated, (c) $320\;\mathrm{mM}H_{2}O_{2}$ + $N_{2}$-saturated, (d) $O_{2}$-saturated, (e) 1mM L-cysteine + air-saturated, (f) 0.2mg/mL SOD + air-saturated, and (g) 0.1M pH 8.0 PBS [183]. The figures were adapted from the cited references.

Competitive immunoassay was constructed by immobilizing HIgG antigen on a meso-2,3-dimercaptosuccinic acid (DMSA)-stabilized CdTe QDs-modified GCE, followed by incubation with different concentrations of HIgG and HRP–anti-HIgG (Figure 9A(a)) [178]. The voltammogram of the QDs/chitosan–HIgG/HRP–anti-HIgG-modified GCE in air-saturated Tris–HCl buffer (pH 9.0) shows two reduction peaks corresponding to dissolved oxygen and DMSA–CdTe QDs at −0.66V and −0.80V, respectively (Figure 9A(b)). Through the reaction of $H_{2}O_{2}$-generated ORR intermediates with ${\mathrm{QD}}^{-\circ }$ in (5a), an intense ECL emission from the CdTe QDs is produced. In the presence of hydroquinone (HQ), consumption of $H_{2}O_{2}$ via its enzymatic reduction to water by HRP leads to a quenching effect, as described in (5b).

(5a) $$\begin{array}{cc} {2\mathrm{CdTe}/\mathrm{DMSA}}^{-\circ }+H_{2}O_{2} & ⟶2{\mathrm{OH}}^{-}+{2\mathrm{CdTe}/\mathrm{DMSA}}^{*} \end{array}$$

(5b) $$\begin{array}{cc} \mathrm{HQ}\;+H_{2}O_{2} & \overset{\mathrm{HRP}}{\to }\mathrm{Quinone}+H_{2}O \end{array}$$

Here, the cyclic enzymatic strategy that consumes the self-produced $H_{2}O_{2}$ co-reactant, generated by the reduction of dissolved oxygen, results in highly sensitive detection of HIgG in the competitive format, with a low LOD of 0.01ng/mL over a dynamic range of 0.05ng/mL to 5μg/mL. It is worth noting that, as the quantity of antigen increases, smaller amounts of antibody are deposited on the surface, leading to reduced catalytic activity of the enzyme and, consequently, an increased signal.

The ORR has been used to quantify biomarkers using high-resolution, ultrasensitive ECL. In these reactions, consumption of the ECL co-reactant (i.e., $O_{2}$ reduction) leads to quenching of the ECL emission. Hemin-functionalized nitrogen-doped graphene-catalyzed ORR has been introduced for ultrasensitive detection of CEA through ECL quenching of CdTe quantum dots (Figure 9B(a)) [179]. While the cathodic light emission of the CdTe QDs-modified GCE in air-saturated PBS (0.1 M, pH 8.0) is prominent due to oxygen electroreduction to the co-reactant $H_{2}O_{2}$ (Figure 9B(b), curve a), a sequential decrease in emission is observed for QDs-modified GCEs subsequently modified with ${\mathrm{Ab}}_{2}$ (curve b), NG (curve c), HRP (curve d), hemin@GO (curve e), and hemin@NG-labeled ${\mathrm{Ab}}_{2}$ (curve f) in the presence of 10 ng/mL antigen. The decrease in ECL emission for the HRP-modified electrode is attributed to steric hindrance, whereas the reduced ECL intensities for the carbon-based nanomaterial electrodes arise from enhanced ECL sensitization in the presence of these nanomaterials. To clarify the mechanism, the electroreduction of dissolved oxygen was examined in air-saturated PBS using CV. As shown in Figure 9B(c), the highest electrocatalytic activity toward reduction of dissolved oxygen to $H_{2}$O or ${\mathrm{OH}}^{-}$ is observed for the sandwich-type immunoreaction using the hemin@NG-modified electrode. This arises from the increased hemin loading on NG and from electron donation by nitrogen atoms in NG, which enhances hemin adsorption and facilitates $O_{2}$ reduction by electrocatalysis [180,181].

In comparison to the hemin-modified GCE, which exhibits negligible current in $N_{2}$-saturated solution (Figure 9B(d), curve a), the reduced hemin can be efficiently oxidized by dissolved oxygen under basic conditions, converting $O_{2}$ into ${\mathrm{OH}}^{-}$ and producing an increased reduction peak (Figure 9B(d), curve b). As expected, a substantially larger reduction peak for hemin in $N_{2}$-saturated electrolyte (Figure 9B(d), curve c), along with a strong electroreduction response toward $O_{2}$ in air-saturated solution, is observed on the hemin@NG-modified GCE (Figure 9B(d), curve d). Based on these results, the electrochemical processes can be described by the equations in (6).

(6a) $$\begin{array}{cc} \mathrm{Hemin}[\mathrm{Fe}(\mathrm{III})]@\mathrm{NG}+e^{-} & ⟶\mathrm{Hemin}[\mathrm{Fe}(\mathrm{II})]@\mathrm{NG} \end{array}$$

(6b) $$\begin{array}{cc} 4\mathrm{Hemin}[\mathrm{Fe}(\mathrm{II})]@\mathrm{NG}+O_{2}+2H_{2}O & ⟶\mathrm{Hemin}[\mathrm{Fe}(\mathrm{III})]@\mathrm{NG}+4{\mathrm{OH}}^{-} \end{array}$$

Therefore, during the ORR and its conversion to ${\mathrm{OH}}^{-}$ (6a), the formation of $H_{2}O_{2}$ as the ECL co-reactant is inhibited, leading to quenched ECL emission. As a result, the hemin@NG signal tag is highly effective for the ECL immunoassay. Under optimized conditions, the ECL intensity of the immunosensor decreased with increasing CEA concentration over the range of 0.1pg/mL to 10ng/mL. The detection limit of 24fg/mL is approximately 20-fold lower than those reported for other amplified electrochemical/ECL approaches [157,182].

QDs-based ECL systems can also be enhanced using $O_{2}^{\circ -}$ as the co-reactant generated from the ORR. To further increase QD-based ECL efficiency, functionalized nanomaterials can be used as catalytic substrates to accelerate emission through enzyme-mimicking activity. Delocalization of nitrogen lone-pair electrons in heteroatom-doped nanotubes can change the chemisorption mode of $O_{2}$ from end-on monoatomic adsorption to side-on diatomic adsorption, effectively weakening the O–O bond and facilitating its reduction to the superoxide intermediate $O_{2}^{\circ -}$ in the ORR [180].

Polystyrene sulfonate (PSS)-functionalized nitrogen-doped carbon nanotubes (NCNTs) have been introduced as labels for secondary antibodies (${\mathrm{Ab}}_{2}$) (Figure 9C(a)), serving as a new ECL signal tag to amplify CdS QD-based ECL in a sandwich immunocomplex formed on an ${\mathrm{Ab}}_{1}$-modified GCE. In this strategy, no strong oxidant is required as a co-reactant, since NCNT-catalyzed generation of $O_{2}^{\circ -}$ from dissolved $O_{2}$ provides an efficient ECL pathway [183]. Unlike signal-off approaches, the signal-on ECL immunosensing configuration avoids false positives that typically arise from steric hindrance associated with immunocomplex formation. The high reduction current and corresponding strong ECL intensity of the CdS-modified GCE in $O_{2}$-saturated solution (Figure 9C(b,d), curve d), compared with $N_{2}$-saturated PBS + $320\;\mathrm{mM}H_{2}O_{2}$ (curve c), demonstrate that dissolved $O_{2}$ is a more effective co-reactant for enhancing QD-based ECL emission. These results also confirm that $H_{2}O_{2}$ is not produced from the reduction of $O_{2}$ via a two-electron pathway.

Introducing L-cysteine (Figure 9 curve e), a scavenger of both OH and superoxide radicals, and superoxide dismutase (SOD) (Figure 9 curve f) led to a decrease in the ECL responses. These results confirmed that the ECL intensity originated from species generated by dissolved $O_{2}^{\circ -}$. Additional electron paramagnetic resonance (EPR) experiments also verified the formation of $O_{2}^{\circ -}$ on the CdS-modified GCE. Because of the nonconductive characteristics of BSA, ${\mathrm{Ab}}_{1}$, and CEA on the CdS/chitosan-modified electrode, the ECL intensity was considerably reduced. Interestingly, a significant increase in ECL intensity was observed in the presence of ${\mathrm{Ab}}_{2}$–PNCNTs. This enhancement arose from the high conversion efficiency of dissolved $O_{2}$ to $O_{2}^{\circ -}$ by the PNCNTs. The signal-on ECL response increased with the CEA concentration over a wide range (50pg/mL to 5mg/mL), with a LOD of 2.4pg/mL, demonstrating that the immunoassay possesses excellent practical applicability in clinical diagnosis without the need for deoxygenation.

### 3.6. Ir Based Single Atom Catalysts (SACs)

Self-powered sensing systems (SPSSs) refer to devices or systems capable of generating and storing the necessary energy to perform sensing functions without relying on external power supplies. SPSSs have been extensively studied in biotechnology as innovative detection platforms. Nevertheless, creating a dependable and long-lasting power source for SPSSs—which must harvest energy from surrounding media such as sunlight, water waves, and air currents—remains a significant challenge. In particular, numerous bioelectrochemical fuel cells have been developed for SPSSs, but their low open-circuit potentials are not suitable for accurate detection [184,185].

Several Ir-based single-atom catalysts (Ir-SACs) have been identified as highly efficient catalysts for the ORR, enabling sensitive analyte detection in SPSSs with stable output signals. The Zhu group successfully developed a hierarchically porous Ir-SAC catalyst that exhibits exceptional ORR performance and remarkable long-term durability in neutral electrolytes. Density functional theory (DFT) calculations revealed that Ir-SAC possesses moderate adsorption energies for ORR intermediates, facilitating the four-electron pathway. Notably, this work demonstrated the feasibility of integrating SPSSs with glucose oxidase and Ir-SAC for the accurate detection of glucose [185].

They also synthesized a nanozymatic biofuel cell with SPSS functionality, composed of Ir-SAC as the cathode and Au nanozymes as the anode. This biofuel cell exhibited exceptional sensitivity and specificity toward prostate-specific antigen detection. Experimental studies confirmed that a single iridium atom catalyst shows outstanding catalytic activity for the ORR under neutral pH conditions. When combined with Au nanozymes at the anode, the Ir-SAC biofuel cell outperformed Pt/C-based biofuel cells. This highly active nanozymatic biofuel cell was therefore employed for the ultrasensitive detection of prostate-specific antigen in an SPSS configuration [184]. Finally, Table 3 summarizes ORR-based biosensors that exploit noble-metal nanoparticle electrocatalysis to generate current or ECL signals directly from dissolved $O_{2}$ under air-saturated conditions, eliminating the need for deoxygenation or external reporters. These platforms achieve low pg/mL sensitivity and broad dynamic ranges, demonstrating the effectiveness of ORR as a robust signal-tracing and amplification mechanism in electrochemical and ECL biosensing.

## 4. Water Splitting-Based Electrochemical Detection

### 4.1. Principles and Mechanism of Water Splitting

Water electrolysis, or electrochemical water splitting, is used as an alternative, clean, and sustainable strategy to replace fossil fuels for the production of hydrogen and oxygen. This process involves the combination of the cathodic hydrogen evolution reaction (HER) and the anodic oxygen evolution reaction (OER), as shown in (7) [186,187].

$$\begin{array}{c} \mathrm{Overall}\;\mathrm{reaction}\text{:}\; \end{array}$$

(7a) $$\begin{array}{c} \;H_{2}O\left(l\right)⟶H_{2}\left(g\right)+\frac{1}{2}O_{2}\left(g\right) \end{array}$$

$$\begin{array}{c} \mathrm{Cathodic}\;\mathrm{reaction}\text{:}\; \end{array}$$

(7b) $$\begin{array}{cc} \;2H^{+}+2e^{-} & ⟶H_{2} \end{array}$$

$$\begin{array}{c} \mathrm{Anodic}\;\mathrm{reaction}\text{:}\; \end{array}$$

(7c) $$\begin{array}{cc} \;2H_{2}O\left(l\right) & ⟶O_{2}\left(g\right)+4H^{+}+4e^{-} \end{array}$$

The possibility of electrochemically detecting various biomarkers without the use of acidic solutions as a proton source for HER or relying on stripping voltammetric detection of completely dissolved nanoparticles is particularly appealing. In addition to the obvious safety concerns, the use of strong acidic media introduces an extra step that not only lengthens the analytical workflow but also poses a substantial obstacle for the development of integrated sensing systems, especially those based on lab-on-a-chip or lateral-flow platforms. A further drawback of HER-based biosensing devices is the formation of hydrogen bubbles during the reaction, which can destabilize the signal and impair assay reproducibility. These limitations highlight the need for novel nanoparticle tags that can be readily detected in immunoreaction media—typically saline buffers with neutral pH. In this context, nanoparticles capable of catalyzing the water oxidation reaction represent a promising solution.

### 4.2. Application of Water-Splitting for Electrochemical Signal Tracing

#### 4.2.1. ${\mathrm{IrO}}_{2}$ NPs

In the investigation reported by Merkoçi’s group, chronoamperometry and cyclic voltammetry were employed to examine the electrocatalytic activity of ${\mathrm{IrO}}_{2}$ NPs in neutral media toward the water oxidation reaction [188]. The voltammetric results demonstrate that as the concentration of ${\mathrm{IrO}}_{2}$ NPs increases, the overpotential decreases and the corresponding currents increase. The amount of ${\mathrm{IrO}}_{2}$ NPs on the SPE transducer correlates with the magnitude of the current recorded in chronoamperometric mode when an appropriate oxidative potential is applied. For ApoE detection—a recognized biomarker for Alzheimer’s disease—the researchers first coated magnetic beads with anti-ApoE antibodies and subsequently introduced ${\mathrm{IrO}}_{2}$ NPs conjugated with secondary anti-ApoE antibodies. The same group later applied an immunoassay based on the same principles to identify a flame-retardant compound, BDE-47 (a polybrominated diphenyl ether, PBDE), achieving detection levels in the ppb range, which meet environmental monitoring requirements [189].

#### 4.2.2. Au NPs

**Figure 10:**
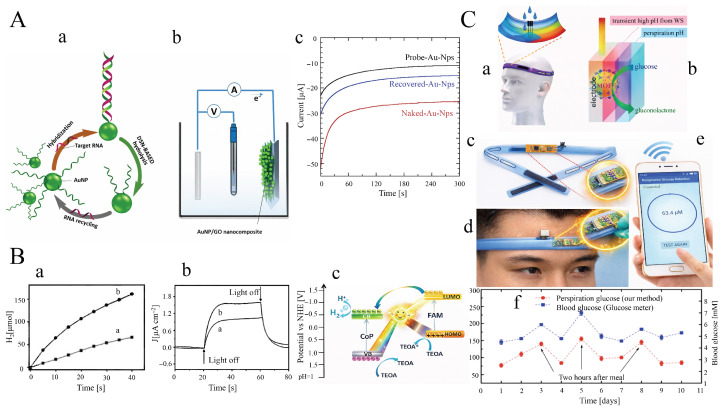
(**A**) (**a**) DSN-assisted target recycling in the presence of Au NPs, (**b**) Electrocatalytic detection using an Au-NP/GO-modified electrode, (**c**) Water-splitting reactions of differently modified electrodes [190]. (**B**) (**a**) $H_{2}$ generation under visible light (λ>420nm) with (a) CoP and (b) PHIV/CoP hybrids, (**b**) Photocurrent of (a) CoP-NWs and (b) PHIV/CoP hybrids measured at a bias of 0.0V vs. Ag/AgCl, (**c**) Mechanism of $H_{2}$ generation using the PHIV/CoP hybrid [193]. (**C**) (**a**) Sweatband glucose sensing, (**b**) Pd@ZIF-67-catalyzed water-splitting electrocatalysis (WSE) for glucose sensing, (**c**) Integration of the sweatband with Pd@ZIF-67, (**d**) Glucose sensing on the sweatband, (**e**) Smartphone-based perspiration analysis, (**f**) Monitoring of glucose concentration in perspiration and blood from a human subject over 10 days [194]. The figures were adapted from the cited references.

The detection of microRNA was demonstrated using a cascade amplification strategy that combined electrocatalytic water-splitting as a signal tracer with duplex-specific nuclease (DSN)-assisted target recycling. The DNA probe was self-assembled onto Au NPs through Au–S chemistry (Figure 10A(a,b)) [190]. In the presence of the microRNA target, the resulting DNA:RNA duplex is specifically hydrolyzed by DSN. During this enzymatic process, the DNA strand is cleaved while the RNA strand remains intact. The surviving RNA target then hybridizes with additional probe DNA strands on the nanoparticle surface, enabling continuous probe digestion and thereby exposing an increasing portion of the Au NP surface. The centrifuge-collected Au NPs were subsequently mixed with graphene oxide (GO) and spin-coated onto the ITO electrode, after which the cathodic amperometric water-splitting reaction was recorded at −1V vs. Ag/AgCl in PBS (1 M, pH 7.4) for 300s. The three-dimensional architecture formed with GO enhances the loading of Au NPs and thus amplifies the signal.

A larger exposed Au NP surface provides more active sites for hydrogen generation, markedly increasing the corresponding current. Water molecules are reduced to ${\mathrm{OH}}^{-}$ and adsorbed hydrogen ($H_{\mathrm{ad}}$) intermediates on the Au NP surface, and $H_{\mathrm{ad}}$ subsequently recombines to produce $H_{2}$ on the Au NP/GO-modified electrode at the applied potential. The electrocatalytic activity of the Au NP–probe/GO–ITO electrode is lower than that of the Au ${\mathrm{NP}}_{\mathrm{naked}}$/GO–ITO electrode (Figure 10A(c)), confirming successful immobilization of the non-conductive DNA probe layer. Upon microRNA hybridization and DSN-assisted recycling, increasingly larger Au NP surface areas become exposed, thereby initiating more efficient cathodic water-splitting catalysis as the probe layer is hydrolyzed. With target microRNA concentrations ranging from 0.1fM to 10pM, the catalytic current progressively increases. Owing to the water-splitting electrocatalytic signal and the recycling amplification mechanism, the proposed Au NP/GO-based biosensor achieves a low LOD of 1.5fM, outperforming other DSN-based microRNA detection approaches that rely on a single amplification step.

#### 4.2.3. Cu(II)-Based Complexs

Caspase-3 is regarded as an essential biological biomarker and therapeutic target in the diagnosis and prognosis of apoptosis-related diseases. In this context, a specific caspase-3 peptide substrate (Ac-GDEVDSK(FFFF)H) is anchored onto a graphene sheet through hydrophobic and π–π interactions [191]. When caspase-3 cleaves the peptide between the D and S residues, the Ac-GDEVD fragment dissociates from the electrode surface, exposing an ATCUN motif (SKH). Consequently, the remaining peptide segment on the electrode is capable of forming a complex with Cu(II), which enhances electrocatalytic water splitting. Unlike existing electrochemical approaches for caspase-3 detection, this signal-on strategy offers a higher signal-to-background ratio and does not require peptide labeling with an electrochemical tracer. Moreover, the close proximity of the ATCUN motif to graphene facilitates electron transfer between Cu(II) and the electrode.

This method enables caspase-3 analysis over a linear range of 0.5pg/mL to 2000pg/mL with a detection limit of 0.2pg/mL. Proteases are enzymes that catalyze peptide bond cleavage, producing shorter peptide fragments. Numerous metal ions are known to bind specific peptide sequences, and protease-mediated cleavage can therefore modulate peptide–metal interactions. Liu’s group investigated the redox properties of various ATCUN–Cu(II) complexes and demonstrated that, under neutral pH conditions, these metallopeptides exhibit strong electrocatalytic activity toward water oxidation. Building on this finding, they developed an immunosensor in which an ${\mathrm{Ab}}_{2}$/protease-modified carbon nanotube (CNT) catalyzes the formation of ATCUN–Cu(II) complexes for water oxidation [192]. In the presence of PSA as a model analyte, the peptide substrate GARGGH is cleaved by trypsin, releasing an ATCUN-containing fragment (GGH) capable of forming a copper complex that catalyzes the water-splitting reaction. PSA concentrations as low as 10pg/mL were detected based on the water electrooxidation activity of the generated ATCUN–Cu(II) complexes.

#### 4.2.4. CoP-Nanowires (CoP-NWs)

The photochemical driving force has been employed as a cost-effective and environmentally sustainable energy source for the sequence-specific detection of DNA using water splitting as an electrochemical tracer [193]. Unlike fluorescence resonance energy transfer (FRET), which requires an external light source and the labeling of sequences with donor–acceptor pairs, photochemical water splitting eliminates these requirements. This feature simplifies and automates the measurement system. Based on the photocatalytic hydrogen generation ability of CoP nanowires (CoP-NWs), the non-covalent attachment of a fluorescein-based dye (FAM)-modified capture probe HIV (PHIV) onto negatively charged CoP-NWs was used for HIV detection.

Upon illumination, an anodic photocurrent was generated by fluorine-doped tin oxide (FTO) modified with CoP-NWs (Figure 10B(a), curve a), indicating that photogenerated electrons were transferred from the n-type CoP-NWs to the FTO electrode. Because the lowest unoccupied molecular orbital (LUMO) level of FAM is more negative than the conduction band (CB) edge of CoP, electron injection from photoexcited FAM into CoP occurs via photoinduced electron transfer (PET), resulting in an enhanced photocurrent on the PHIV/CoP-modified FTO (Figure 10B(a), curve b). The increased photochemical hydrogen evolution under visible light for PHIV/CoP hybrids, compared with CoP-NW- modified FTO in the presence of triethanolamine (TEOA) as a sacrificial agent, further confirms that high electron accumulation in the CB of CoP-NWs originates from PET from the excited dye’s LUMO (Figure 10B(b)).

With increasing concentrations of the complementary HIV target, the affinity of CoP-NWs toward the resulting double-stranded DNA (dsDNA) decreases due to the absence of mismatched nucleobases. Consequently, electron accumulation in CoP-NWs is reduced because electron transfer from FAM to the CB of CoP-NWs is hindered. This process leads to a decrease in photocatalytic $H_{2}$ generation from water as the target concentration increases (Figure 10B(c)). The good electrical conductivity of CoP-NWs, combined with the strong photocatalytic $H_{2}$ generation in the absence of any electrochemical signal tracer, makes this strategy highly suitable for sensitive point-of-care detection.

#### 4.2.5. Pd-Based NPs

Wearable perspiration glucose sensing is being developed using an enzyme-free electrochemical sensor based on a water-splitting signal. Unlike previously reported nonenzymatic glucose sensors that detect glucose in alkaline buffers, Zhu et al. [194] proposed a glucose sensor operating at physiological pH without the need for additional reagents, enabling long-term, wearable, and maintenance-free glucose monitoring. In this work, a screen-printed Pd nanoparticle-encapsulated Co-based zeolitic imidazolate framework (Pd@ZIF-67) carbon conductive ink on a flexible PET substrate was employed for highly sensitive and reproducible detection of glucose in perspiration using a water-splitting-assisted electrocatalytic (WSE) reaction (Figure 10C). In this regard, a multistep potential protocol versus PVA/KCl–Ag/AgCl was applied to the electrode, including a high negative potential (−2.0V) to generate localized alkalinity via electrochemical water splitting, a moderate potential (+0.6V) to oxidize glucose through the conversion of [Co(III)(mim)_2_(OH)]_*n*_/[Co(IV)(mim)_2_(OH)_2_]_*n*_, and a highly positive potential (+1.0V) to regenerate the electrode:

$$2H_{2}O+2e^{-}\overset{\;-2.0\;V\;}{\to }H_{2}+2{\mathrm{OH}}^{-}$$

Application of a high negative potential generates hydrogen bubbles, which can introduce uncertainty into glucose measurement. In this study, this challenge was mitigated using Pd@ZIF-67: the “spillover” characteristics of Pd NPs embedded in ZIF-67 catalytically dissociate the generated $H_{2}$ into monatomic hydrogen, which subsequently combines with the Pd matrix [195,196,197]. The wearable sweatband incorporating Pd@ZIF-67 enabled nonenzymatic glucose detection in perspiration with an acceptable shelf life (2 months under ambient conditions), a linear response from 10 μM to 1000 μM, and a limit of detection of 2.0 μM.

Table 4 provides a comparative overview of water-splitting and $H_{2}O_{2}$-based electrochemical biosensors, summarizing key experimental conditions, signal readout modes, and reported analytical performance.

While the preceding sections discuss representative case studies for HER-, ORR-, and water-splitting-based biosensing strategies, cross-comparison of analytical performance across different signal transduction approaches remains challenging due to variations in assay formats and experimental conditions. To provide quantitative context, it is therefore instructive to benchmark energy-based electrocatalytic biosensing strategies against conventional redox-label and enzyme-based electrochemical techniques at the order-of-magnitude level.

Accordingly, Table 5 summarizes the overall ranges of LOD and linear/dynamic ranges reported for energy-based electrocatalytic biosensing strategies and compares them with traditional redox-label and enzyme-based electrochemical techniques. The reported values are extracted from representative recent studies and reviews and are intended to reflect typical order-of-magnitude performance observed across different analytes, matrices, and assay formats, rather than direct head-to-head experimental comparisons. Overall, this comparison indicates that energy-based electrocatalytic biosensing strategies can achieve sensitivities and dynamic ranges comparable, at the order-of-magnitude level, to conventional redox-label and enzyme-based electrochemical techniques, while offering additional advantages such as reagentless operation and simplified assay design.

## 5. Conclusions

This study’s main purpose is to provide a comprehensive, authoritative, and essential resource for researchers on the interdisciplinary between energy and biosensors. Research progress into the desire for new signal recording pathways using the versatility of the HER/ORR and water splitting during the last few decades is highlighted. Current challenges in the field of biosensors are presented together with strategies to overcome these obstacles; one strategy is using energy-to-biosensor conversion. Another goal of this study is to bridge the gap between biosensors and commercialization, which is currently one of the “hottest” challenges in the field of POC bioassays. As indicated in this review, the increase in the introduction of new materials for recording the signal in only supporting electrolytes without any electroactive labels has been particularly encouraging to date.

The field of HER/ORR/water splitting-based materials for signal recording in reagentless and electrochemical redox reporter-free biosensors is very promising. However, there are several challenges and unanswered questions that require further exploration in future research. It is essential to continue investigating and addressing these issues to fully realize the potential of these materials in biosensor applications.

In particular, the reproducibility of catalytic biosensors—especially those employing nanoparticle labels—remains a critical issue, as batch-to-batch variability and surface heterogeneity can significantly affect signal consistency and hinder reliable translation. Second, using both crystalline and amorphous structures to capture signals has proven to be an effective method for achieving exceptional material performance. The enhanced catalytic activity and strengthening effect stem from the distinctive atomic arrangements. Hence, it is crucial to design nanomaterials with unique structures for the use of HER/ORR/water splitting to comprehend the relationship between the structure and properties of nanomaterials. However, conducting a precise structural analysis is challenging because of the difficulty in accurately determining the atomic arrangement in both amorphous and crystalline structures, as well as at phase interfaces. A second factor influencing the durability of nanostructures is their thermal stability, which has hardly been investigated in engineering applications. In fact, the applications of these nanoparticles at human body temperature and above to fight diseases should be investigated. Also, creating flexible and stretchable biosensors may appear to be a viable solution, but it may not be feasible for devices that require a steady power source for ongoing monitoring of physiological functions. In this context, generating power from the dynamic micromotions of implanted tissues and the human body is highly desirable for sustaining device functionality. However, substantial progress is needed to obtain sufficient energy to power soft functional devices. Finally, seeking nanoparticles that can function properly in biological pH conditions is another challenge that should be considered.

## Figures and Tables

**Table 1:** Summary of HER-based electrochemical biosensors discussed in this section. NR: not reported explicitly in the cited work.

Target (Label)	Assay	Electrolyte	Readout	Condition	LOD	Range
Rabbit IgG(${\mathrm{WS}}_{2}$)	Magneto-sandwich	$0.5\;MH_{2}{\mathrm{SO}}_{4}$	EIS	−0.57–−0.77 V (opt. −0.77 V)	2ng/mL	2–500 ng/mL
Rabbit IgG(${\mathrm{MoSe}}_{2}$)	Competitive magneto	$0.5\;MH_{2}{\mathrm{SO}}_{4}$	CA (200 s)	−0.97 V	1.23ng/mL	2–500 ng/mL
Rabbit IgG(BP)	Competitive(nanoimpact)	$1\;MH_{2}{\mathrm{SO}}_{4}$	CA (40 s)	−0.88 V	0.98ng/mL	2–100 ng/mL
PSA(BE–ECL; Ru)	Sandwich(BE–ECL)	Cathode:$0.5\;MH_{2}{\mathrm{SO}}_{4}$	ECL Smartphonereadout	$\eta_{10}=-114$ mV ($81\;{\mathrm{mV dec}}^{-1}$)	0.1pg/mL	NR
hIgG (Pt; Cu inhib.)	Sandwich ELEIA	GL–NaOH(pH 10)0.1M HCl	Chronopot.	500 μA	1.0pg/mL	1–100 pg/mL
TC (PtGN)	Competitive + Pt growth	10mM HCl + 1M KCl	CV/LSV	∼−0.42 V (Pt growth)	6pg/mL	0.05–100 ng/mL
HIgG (Au NPs)	Magneto-sandwich	1M HCl	CA	+1.35V (pre-ox) −1.0 V	1.45ng/mL	5–1000 ng/mL
CA19-9 (Au–PAAD)	Sandwich	2M HCl	CA	+1.35V/−1.0 V (50 s)	6.3mU/mL	0.01–300 U/mL
IgG(Au; EIS)	Immunoassay(Au tag)	2M HCl	EIS	+1.35Vpre-step;DC bias	0.31ng/mL	2–500 ng/mL

References: [111,112,114,120,125,131,135,147,148].

**Table 2:** ORR processes with their thermodynamic potentials under standard conditions. ^a,b^: Because the potentials for the one-electron reduction of oxygen to $O_{2}^{-}$ and subsequently to $O_{2}^{2-}$ are strongly influenced by the solvent, their exact values are not included in the table. TP denotes the thermodynamic potential.

Electrolyte	ORR Reaction	TP [V]
Acidic aqueous solution	$O_{2}+4H^{+}+4e^{-}⟶H_{2}O$	1.229V
	$O_{2}+4H^{+}+2e^{-}⟶H_{2}O_{2}$	0.7V
	$H_{2}O_{2}+2H^{+}+2e^{-}⟶2H_{2}O$	1.76V
Alkaline aqueous solution	$O_{2}+H_{2}O+4e^{-}⟶4{\mathrm{OH}}^{-}$	0.401V
	$O_{2}+H_{2}O+2e^{-}⟶{\mathrm{OH}}^{-}+{\mathrm{HO}}_{2}^{-}$	−0.065V
	${\mathrm{HO}}_{2}^{-}+H_{2}O+2e^{-}⟶3{\mathrm{OH}}^{-}$	0.867V
Neutral aqueous solution	$O_{2}+2H_{2}O+4e^{-}⟶H_{2}O_{2}+2{\mathrm{OH}}^{-}$	−0.867V
Non-aqueous aprotic solvents	$O_{2}+e^{-}⟶O_{2}^{-}$	a
	$O_{2}+e^{-}⟶O_{2}^{-}$	b

**Table 3:** Summary of ORR-based electrochemical biosensors discussed in this section. NR: not reported in the cited work within the present discussion.

Item	HIgG(PtNDs@GS)	HIgG(PdNP–CNT Labels)	CEA(Pd NPs@PAMAM/SWNH)
Assay format	Sandwich immunoassay (Ab tag)	Sandwich immunosensor (PdNP–Ab label)	Sandwich ECL assay (QD, signal-off)
Electrolyte (pH)	Air-saturated PBS (7.4)	Air-saturated electrolyte(NR pH)	Air-saturated PBS (9.0)
Readout mode	DPV (ORR tracer current)	ORR-based current signal	ECL (ORR quenching; ${\mathrm{4e}}^{-}$)
LOD	NR	40pg/mL	0.47pg/mL
Linear range	1pg/mL–10ng/mL	50pg/mL–10ng/mL	1pg/mL–100ng/mL
Key interference	No deoxygenation; negligible in $N_{2}$-saturated PBS	No reporter; air operation	Air operation; ECL quenched by ORR
Reference	[155]	[156]	[157]

**Table 4:** Representative electrochemical biosensors exploiting water-splitting reactions (HER, OER, or photocatalytic splitting) as signal tracers.

Target Analyte	Electrocatalyst	Water-Splitting Mechanism	Electrolyte (pH)	LOD
ApoE/BDE-47	${\mathrm{IrO}}_{2}$ NPs	Anodic water oxidation (OER)	Neutral buffer	NR
miRNA	Au NPs/GO	Cathodic water reduction (HER)	PBS (7.4)	1.5fM
Caspase-3	ATCUN–Cu(II) complex	Water oxidation catalysis	Neutral buffer	0.2pg/mL
DNA (HIV)	CoP nanowires	Photocatalytic $H_{2}$ generation	Aqueous medium	NR
Glucose (perspiration)	Pd@ZIF-67	Water-splitting–assisted electrocatalysis	Physiological pH	2.0 μM

**Table 5:** Order-of-magnitude quantitative benchmarking of energy-based electrocatalytic biosensing strategies compared with conventional redox-label and enzyme-based electrochemical techniques.

Approach	Overall LOD Range (Reported)	Overall Linear/Dynamic Range(Reported)	References
Energy-based electrocatalytic readouts(HER/ORR/OER)	fg/mL → pg/mL	Typically ∼3–6 orders of magnitude (e.g., 0.001–100 ng/mL)	[198,199]
Redox-label/redox-reporter techniques	∼0.1 pg/mL → several pg/mL	Typically ∼4–6 orders of magnitude	[86,200,201,202]
Enzyme-based electrochemical techniques (HRP/ALP)	fg/mL → pg/mL	Typically ∼3–6 orders of magnitude	[203,204,205,206,207]

## Data Availability

No new data were generated or analysed in this study. All data discussed in this review are available in the cited literature.

## Abbreviations

The following abbreviations are used in this manuscript:

**Abbrev.**	**Description**
AFP	Alpha-fetoprotein
LSV	Linear sweep voltammetry
CA15-3	Cancer antigen 15-3
ORR	Oxygen reduction reaction
CA19-9	Cancer antigen 19-9
PBS	Phosphate-buffered saline
CEA	Carcinoembryonic antigen
QDs	Quantum dots
CNTs	Carbon nanotubes
SPE	Screen-printed electrode
CV	Cyclic voltammetry
HER	Hydrogen evolution reaction
DPV	Differential pulse voltammetry
HIgG	Human immunoglobulin G
ECL	Electrochemiluminescence
LOD	Limit of detection
EIS	Electrochemical impedance spectroscopy
GCE	Glassy carbon electrode
SPCE	Screen-printed carbon electrode
MB	Methylene blue
BE	Bipolar electrochemistry
TMDs	Transition-metal dichalcogenides
PAMAM	poly(amidoamine)

## References

[1] Mayer K.M., Hafner J.H. (2011). Localized surface plasmon resonance sensors. Chem. Rev..

[2] Hou W., Cronin S.B. (2013). A review of surface plasmon resonance-enhanced photocatalysis. Adv. Funct. Mater..

[3] Homola J. (2003). Present and future of surface plasmon resonance biosensors. Anal. Bioanal. Chem..

[4] Wang Q., Ren Z.-H., Zhao W.-M., Wang L., Yan X., Zhu A.-S., Qiu F.-M., Zhang K.-K. (2022). Research advances on surface plasmon resonance biosensors. Nanoscale.

[5] Azzouz A., Hejji L., Kim K.-H., Kukkar D., Souhail B., Bhardwaj N., Brown R.J.C., Zhang W. (2022). Advances in surface plasmon resonance–based biosensor technologies for cancer biomarker detection. Biosens. Bioelectron..

[6] Chen T., Xin J., Chang S.J., Chen C.-J., Liu J.-T. (2023). Surface plasmon resonance (SPR) combined technology: A powerful tool for investigating interface phenomena. Adv. Mater. Interfaces.

[7] Khoshfetrat S.M., Khodadadian A., Mirsian S., Hilber W., Heitzinger C. (2025). The marriage between energy and biosensor. ChemRxiv.

[8] Lim H.J., Saha T., Tey B.T., Tan W.S., Ooi C.W. (2020). Quartz crystal microbalance-based biosensors as rapid diagnostic devices for infectious diseases. Biosens. Bioelectron..

[9] Cheng C.I., Chang Y.-P., Chu Y.-H. (2012). Biomolecular interactions and tools for their recognition: Focus on the quartz crystal microbalance and its diverse surface chemistries and applications. Chem. Soc. Rev..

[10] Shu W., Laurenson S., Knowles T.P.J., Ferrigno P.K., Seshia A.A. (2008). Highly specific label-free protein detection from lysed cells using internally referenced microcantilever sensors. Biosens. Bioelectron..

[11] Hansen K.M., Thundat T. (2005). Microcantilever biosensors. Methods.

[12] Boisen A., Thundat T. (2009). Design & fabrication of cantilever array biosensors. Mater. Today.

[13] Chakraborty A., Dutta P., Wakankar A., RoyChaudhuri C. (2022). Recent field effect transistors and electrical impedance spectroscopy based biosensing strategies for cancer biomarker screening: A mini review. Biosens. Bioelectron. X.

[14] Strong M.E., Richards J.R., Torres M., Beck C.M., La Belle J.T. (2021). Faradaic electrochemical impedance spectroscopy for enhanced analyte detection in diagnostics. Biosens. Bioelectron..

[15] Magar H.S., Hassan R.Y.A., Mulchandani A. (2021). Electrochemical impedance spectroscopy (EIS): Principles, construction, and biosensing applications. Sensors.

[16] Shkodra B., Petrelli M., Angeli M.A.C., Garoli D., Nakatsuka N., Lugli P., Petti L. (2021). Electrolyte-gated carbon nanotube field-effect transistor-based biosensors: Principles and applications. Appl. Phys. Rev..

[17] Krishnan S.K., Nataraj N., Meyyappan M., Pal U. (2023). Graphene-based field-effect transistors in biosensing and neural interfacing applications: Recent advances and prospects. Anal. Chem..

[18] Sadighbayan D., Hasanzadeh M., Ghafar-Zadeh E. (2020). Biosensing based on field-effect transistors (FET): Recent progress and challenges. TrAC Trends Anal. Chem..

[19] Novodchuk I., Bajcsy M., Yavuz M. (2021). Graphene-based field effect transistor biosensors for breast cancer detection: A review on biosensing strategies. Carbon.

[20] Mirsian S., Khodadadian A., Hedayati M., Manzour-ol-Ajdad A., Kalantarinejad R., Heitzinger C. (2019). A new method for selective functionalization of silicon nanowire sensors and Bayesian inversion for its parameters. Biosens. Bioelectron..

[21] Stern E., Klemic J.F., Routenberg D.A., Wyrembak P.N., Turner-Evans D.B., Hamilton A.D., LaVan D.A., Fahmy T.M., Reed M.A. (2007). Label-free immunodetection with CMOS-compatible semiconducting nanowires. Nature.

[22] Cui Y., Wei Q., Park H., Lieber C.M. (2001). Nanowire nanosensors for highly sensitive and selective detection of biological and chemical species. Science.

[23] Mirsian S., Hilber W., Khodadadian E., Parvizi M., Khodadadian A., Khoshfetrat S.M., Heitzinger C., Jakoby B. (2025). Graphene-based FETs for advanced biocatalytic profiling: Investigating heme peroxidase activity with machine learning insights. Microchim. Acta.

[24] Khodadadian E., Mirsian S., Shashaani S., Parvizi M., Khodadadian A., Knees P., Hilber W., Heitzinger C. (2025). A Bayesian inversion supervised learning framework for the enzyme activity in graphene field-effect transistors. Mach. Learn. Appl..

[25] Khodadadian E., Goldoni D., Nicolini J., Khodadadian A., Heitzinger C., Selmi L. (2025). Integrating physics-based simulations, machine learning, and Bayesian inference for accurate detection and metrology of elongated nanoscale analytes using high-frequency capacitance spectroscopy. Eng. Appl. Artif. Intell..

[26] Khodadadian A., Parvizi M., Teshnehlab M., Heitzinger C. (2022). Rational design of field-effect sensors using partial differential equations, Bayesian inversion, and artificial neural networks. Sensors.

[27] Fägerstam L.G., Frostell Å, Karlsson R., Kullman M., Anita L., Malmqvist M., Butt H. (1990). Detection of antigen–antibody interactions by surface plasmon resonance. Application to epitope mapping. J. Mol. Recognit..

[28] Fivash M., Towler E.M., Fisher R.J. (1998). BIAcore for macromolecular interaction. Curr. Opin. Biotechnol..

[29] Rich R.L., Myszka D.G. (2008). Survey of the year 2007 commercial optical biosensor literature. J. Mol. Recognit. Interdiscip. J..

[30] Pumera M., Sanchez S., Ichinose I., Tang J. (2007). Electrochemical nanobiosensors. Sens. Actuators B Chem..

[31] Ghaffari R., Choi J., Raj M.S., Chen S., Lee S.P., Reeder J.T., Aranyosi A.J., Leech A., Li W., Schon S. (2020). Soft wearable systems for colorimetric and electrochemical analysis of biofluids. Adv. Funct. Mater..

[32] Parrilla M., Wael K.D. (2021). Wearable self-powered electrochemical devices for continuous health management. Adv. Funct. Mater..

[33] Eliaz N., Gileadi E. (2019). Physical Electrochemistry: Fundamentals, Techniques, and Applications.

[34] Wang S., Zhang J., Gharbi O., Vivier V., Gao M., Orazem M.E. (2021). Electrochemical impedance spectroscopy. Nat. Rev. Methods Prim..

[35] Lakshmi K.C.S., Vedhanarayanan B., Lin T.-W. (2023). Electrocatalytic hydrogen and oxygen evolution reactions: Role of two-dimensional layered materials and their composites. Electrochim. Acta.

[36] Hai X., Li Y., Zhu C., Song W., Cao J., Bi S. (2020). Dna-based label-free electrochemical biosensors: From principles to applications. TrAC Trends Anal. Chem..

[37] Hu Y., Shen Q., Li W., Liu Z., Nie Z., Yao S. (2015). A TdT-mediated cascade signal amplification strategy based on dendritic DNA matrix for label-free multifunctional electrochemical biosensing. Biosens. Bioelectron..

[38] Wang M., Fu Z., Li B., Zhou Y., Yin H., Ai S. (2014). One-step, ultrasensitive, and electrochemical assay of microRNAs based on T7 exonuclease assisted cyclic enzymatic amplification. Anal. Chem..

[39] Liu S., Liu T., Wang L. (2015). Label-free, isothermal and ultrasensitive electrochemical detection of DNA and DNA 3’-phosphatase using a cascade enzymatic cleavage strategy. Chem. Commun..

[40] Liu L., Xia N., Liu H., Kang X., Liu X., Xue C., He X. (2014). Highly sensitive and label-free electrochemical detection of microRNAs based on triple signal amplification of multifunctional gold nanoparticles, enzymes and redox-cycling reaction. Biosens. Bioelectron..

[41] Khoshfetrat S.M., Mehrgardi M.A. (2014). Dual amplification of single nucleotide polymorphism detection using graphene oxide and nanoporous gold electrode platform. Analyst.

[42] Wongkaew N., Simsek M., Griesche C., Baeumner A.J. (2018). Functional nanomaterials and nanostructures enhancing electrochemical biosensors and lab-on-a-chip performances: Recent progress, applications, and future perspective. Chem. Rev..

[43] Khoshfetrat S.M., Ranjbari M., Shayan M., Mehrgardi M.A., Kiani A. (2015). Wireless electrochemiluminescence bipolar electrode array for visualized genotyping of single nucleotide polymorphism. Anal. Chem..

[44] Lin D., Wu J., Wang M., Yan F., Ju H. (2012). Triple signal amplification of graphene film, polybead carried gold nanoparticles as tracing tag and silver deposition for ultrasensitive electrochemical immunosensing. Anal. Chem..

[45] Khoshfetrat S.M., Sheini A. (2026). CTAB-assisted interfacing of Co LDH nanosheets with in situ MXene-derived TiO_2_ for enhanced oxygen evolution reaction. Fuel.

[46] Khoshfetrat S.M., Motahari M., Mirsian S. (2025). 3D porous structure of ionic liquid-delaminated Ti3C2 MXene nanosheets for enhanced electrochemical sensing of tryptophan in real samples. Sci. Rep..

[47] Dequaire M., Degrand C., Limoges B. (2000). An electrochemical metalloimmunoassay based on a colloidal gold label. Anal. Chem..

[48] Liang W., Wied P., Carraro F., Sumby C.J., Nidetzky B., Tsung C.-K., Falcaro P., Doonan C.J. (2021). Metal–organic framework-based enzyme biocomposites. Chem. Rev..

[49] Jiang X., Wang Z., Wang H., Zhuo Y., Yuan R., Chai Y. (2017). A novel metal–organic framework loaded with abundant N-(aminobutyl)-N-(ethylisoluminol) as a high-efficiency electrochemiluminescence indicator for sensitive detection of mucin1 on cancer cells. Chem. Commun..

[50] Ahmadi A., Khoshfetrat S.M., Kabiri S., Dorraji P.S., Larijani B., Omidfar K. (2021). Electrochemiluminescence paper-based screen-printed electrode for HbA1c detection using two-dimensional zirconium metal-organic framework/Fe_3_O_4_ nanosheet composites decorated with Au nanoclusters. Microchim. Acta.

[51] Khoshfetrat S.M., Hashemi P., Afkhami A., Hajian A., Bagheri H. (2021). Cascade electrochemiluminescence-based integrated graphitic carbon nitride-encapsulated metal-organic framework nanozyme for prostate-specific antigen biosensing. Sens. Actuators B Chem..

[52] Wang C., Liao K. (2021). Recent advances in emerging metal–and covalent–organic frameworks for enzyme encapsulation. ACS Appl. Mater. Interfaces.

[53] Uttam B., Kumar R. (2021). Metal-organic frameworks as sensors. Metal-Organic Frameworks for Environmental Sensing.

[54] Liu J., Goetjen T.A., Wang Q., Knapp J.G., Wasson M.C., Yang Y., Syed Z.H., Delferro M., Notestein J.M., Farha O.K. (2022). MOF-enabled confinement and related effects for chemical catalyst presentation and utilization. Chem. Soc. Rev..

[55] Lian X., Fang Y., Joseph E., Wang Q., Li J., Banerjee S., Lollar C., Wang X., Zhou H.-C. (2017). Enzyme–MOF (metal–organic framework) composites. Chem. Soc. Rev..

[56] Chen W.-H., Yu X., Liao W.-C., Sohn Y.S., Cecconello A., Kozell A., Nechushtai R., Willner I. (2017). ATP-Responsive aptamer-based metal–organic framework nanoparticles (NMOFs) for the controlled release of loads and drugs. Adv. Funct. Mater..

[57] Khoshfetrat S.M. (2025). In situ synthesis of nickel-substituted zeolitic metal-organic framework on Ti3C2 MXene for enhanced electrocatalytic sensing of L-tryptophan. J. Electroanal. Chem..

[58] Wang S., McGuirk C.M., d’Aquino A., Mason J.A., Mirkin C.A. (2018). Metal–organic framework nanoparticles. Adv. Mater..

[59] Liao X., Fu H., Yan T., Lei J. (2019). Electroactive metal–organic framework composites: Design and biosensing application. Biosens. Bioelectron..

[60] Liu J., Liu T., Du P., Zhang L., Lei J. (2019). Metal–organic framework (MOF) hybrid as a tandem catalyst for enhanced therapy against hypoxic tumor cells. Angew. Chem..

[61] Li W.-H., Ding K., Tian H.-R., Yao M.-S., Nath B., Deng W.-H., Wang Y., Xu G. (2017). Conductive metal–organic framework nanowire array electrodes for high-performance solid-state supercapacitors. Adv. Funct. Mater..

[62] Wang J., Liu G., Wu H., Lin Y. (2008). Quantum-dot-based electrochemical immunoassay for high-throughput screening of the prostate-specific antigen. Small.

[63] Cai H., Xu Y., Zhu N., He P., Fang Y. (2002). An electrochemical DNA hybridization detection assay based on a silver nanoparticle label. Analyst.

[64] Wang J., Xu D., Kawde A.-N., Polsky R. (2001). Metal nanoparticle-based electrochemical stripping potentiometric detection of DNA hybridization. Anal. Chem..

[65] Pollok N.E., Rabin C., Smith L., Crooks R.M. (2019). Orientation-controlled bioconjugation of antibodies to silver nanoparticles. Bioconjug. Chem..

[66] Scida K., Cunningham J.C., Renault C., Richards I., Crooks R.M. (2014). Simple, sensitive, and quantitative electrochemical detection method for paper analytical devices. Anal. Chem..

[67] Cunningham J.C., Scida K., Kogan M.R., Wang B., Ellington A.D., Crooks R.M. (2015). Paper diagnostic device for quantitative electrochemical detection of ricin at picomolar levels. Lab Chip.

[68] Lubin A.A., Plaxco K.W. (2010). Folding-based electrochemical biosensors: The case for responsive nucleic acid architectures. Acc. Chem. Res..

[69] Ma M., Miao Z., Zhang D., Du X., Zhang Y., Zhang C., Lin J., Chen Q. (2015). Highly-ordered perpendicularly immobilized FWCNTs on the thionine monolayer-modified electrode for hydrogen peroxide and glucose sensors. Biosens. Bioelectron..

[70] Gao F., Gao C., He S., Wang Q., Wu A. (2016). Label-free electrochemical lead (II) aptasensor using thionine as the signaling molecule and graphene as signal-enhancing platform. Biosens. Bioelectron..

[71] Cui L., Wang M., Sun B., Ai S., Wang S., Zhang C.-Y. (2019). Substrate-free and label-free electrocatalysis-assisted biosensor for sensitive detection of microRNA in lung cancer cells. Chem. Commun..

[72] Beitollahi H., Khalilzadeh M.A., Tajik S., Safaei M., Zhang K., Jang H.W., Shokouhimehr M. (2020). Recent advances in applications of voltammetric sensors modified with ferrocene and its derivatives. ACS Omega.

[73] Fabre B. (2010). Ferrocene-terminated monolayers covalently bound to hydrogen-terminated silicon surfaces. Toward the development of charge storage and communication devices. Acc. Chem. Res..

[74] Ge L., Wang W., Li F. (2017). Electro-grafted electrode with graphene-oxide-like DNA affinity for ratiometric homogeneous electrochemical biosensing of microRNA. Anal. Chem..

[75] Wu L., Ji H., Sun H., Ding C., Ren J., Qu X. (2016). Label-free ratiometric electrochemical detection of the mutated apolipoprotein E gene associated with Alzheimer’s disease. Chem. Commun..

[76] Ikeda R., Kitagawa S., Chiba J., Inouye M. (2009). Electrochemical Genotyping by Using Two Ferrocene/Isoquinoline-Connected DNA Probes with Different Redox Potentials on a Single Electrode. Chem.-Eur. J..

[77] Fan C., Plaxco K.W., Heeger A.J. (2003). Electrochemical interrogation of conformational changes as a reagentless method for the sequence-specific detection of DNA. Proc. Natl. Acad. Sci. USA.

[78] Mie Y., Kowata K., Kojima N., Komatsu Y. (2012). Electrochemical properties of interstrand cross-linked DNA duplexes labeled with Nile blue. Langmuir.

[79] Du P., Liu S., Wu P., Cai C. (2007). Single-walled carbon nanotubes functionalized with poly (nile blue A) and their application to dehydrogenase-based biosensors. Electrochim. Acta.

[80] Gorodetsky A.A., Hammond W.J., Hill M.G., Slowinski K., Barton J.K. (2008). Scanning electrochemical microscopy of DNA monolayers modified with Nile Blue. Langmuir.

[81] Feng C., Ma L., Li F., Mai H., Lang X., Fan S. (2010). A polypyrrole/anthraquinone-2, 6-disulphonic disodium salt (PPy/AQDS)-modified anode to improve performance of microbial fuel cells. Biosens. Bioelectron..

[82] Balintová J., Pohl R., Horáková P., Vidláková P., Havran L., Fojta M., Hocek M. (2011). Anthraquinone as a Redox Label for DNA: Synthesis, Enzymatic Incorporation, and Electrochemistry of Anthraquinone-Modified Nucleosides, Nucleotides, and DNA. Chem.-Eur. J..

[83] Pheeney C.G., Barton J.K. (2013). Intraduplex DNA-mediated electrochemistry of covalently tethered redox-active reporters. J. Am. Chem. Soc..

[84] Long D., Li M., Wang H., Wang H., Chai Y., Yuan R. (2019). A photoelectrochemical biosensor based on fullerene with methylene blue as a sensitizer for ultrasensitive DNA detection. Biosens. Bioelectron..

[85] Chang J., Lv W., Li Q., Li H., Li F. (2020). One-step synthesis of methylene blue-encapsulated zeolitic imidazolate framework for dual-signal fluorescent and homogeneous electrochemical biosensing. Anal. Chem..

[86] Kang D., Ricci F., White R.J., Plaxco K.W. (2016). Survey of redox-active moieties for application in multiplexed electrochemical biosensors. Anal. Chem..

[87] Ghica M.E., Brett C.M.A. (2008). Glucose oxidase inhibition in poly (neutral red) mediated enzyme biosensors for heavy metal determination. Microchim. Acta.

[88] Hirano K., Yamato H., Kunimoto K., Ohwa M. (2002). Design of novel electron transfer mediators based on indophenol derivatives for lactate sensor. Biosens. Bioelectron..

[89] Liu X., Zhang H., Song Z., Guo L., Fu F., Wu Y. (2019). A ratiometric nanoprobe for biosensing based on green fluorescent graphitic carbon nitride nanosheets as an internal reference and quenching platform. Biosens. Bioelectron..

[90] Hong C.-Y., Zhang X.-X., Dai C.-Y., Wu C.-Y., Huang Z.-H. (2020). Highly sensitive detection of multiple antibiotics based on DNA tetrahedron nanostructure-functionalized magnetic beads. Anal. Chim. Acta.

[91] Khoshfetrat S.M., Mehrgardi M.A. (2014). Electrochemical genotyping of single-nucleotide polymorphisms by using monobase-conjugated modified nanoparticles. ChemElectroChem.

[92] Yang Y., Gu Y., Wan B., Ren X., Guo L.-H. (2017). Label-free electrochemical biosensing of small-molecule inhibition on O-GlcNAc glycosylation. Biosens. Bioelectron..

[93] Boon E.M., Barton J.K. (2002). Charge transport in DNA. Curr. Opin. Struct. Biol..

[94] Mehrgardi M.A., Daneshtalab R. (2011). Electrochemical detection of different types of single-base mismatches in DNA using copper-phthalocyanine tetrasulfonic acid. J. Electroanal. Chem..

[95] Kelley S.O., Barton J.K., Jackson N.M., Hill M.G. (1997). Electrochemistry of methylene blue bound to a DNA-modified electrode. Bioconjug. Chem..

[96] Kelley S.O., Boon E.M., Barton J.K., Jackson N.M., Hill M.G. (1999). Single-base mismatch detection based on charge transduction through DNA. Nucleic Acids Res..

[97] Wong E.L.S., Gooding J.J. (2003). Electronic detection of target nucleic acids by a 2, 6-disulfonic acid anthraquinone intercalator. Anal. Chem..

[98] Pimpilova M. (2024). A brief review on methods and materials for electrode modification: Electroanalytical applications towards biologically relevant compounds. Discov. Electrochem..

[99] Tricase A., Muhyuddin M., Erable B., Atanassov P., Pant D., Santoro C., Bollella P. (2025). Bio-and electrocatalysts for oxygen reduction reaction in neutral media: From mechanisms to practical applications. J. Power Sources.

[100] Zhu J., Hu L., Zhao P., Lee L.Y.S., Wong K.-Y. (2019). Recent advances in electrocatalytic hydrogen evolution using nanoparticles. Chem. Rev..

[101] El-Refaei S.M., Russo P.A., Pinna N. (2021). Recent advances in multimetal and doped transition-metal phosphides for the hydrogen evolution reaction at different pH values. ACS Appl. Mater. Interfaces.

[102] Wang Q.H., Kalantar-Zadeh K., Kis A., Coleman J.N., Strano M.S. (2012). Electronics and optoelectronics of two-dimensional transition metal dichalcogenides. Nat. Nanotechnol..

[103] Kalantar-Zadeh K., Ou J.Z., Daeneke T., Strano M.S., Pumera M., Gras S.L. (2015). Two-dimensional transition metal dichalcogenides in biosystems. Adv. Funct. Mater..

[104] Li S., Wang S., Tang D.-M., Zhao W., Xu H., Chu L., Bando Y., Golberg D., Eda G. (2015). Halide-assisted atmospheric pressure growth of large WSe2 and WS2 monolayer crystals. Appl. Mater. Today.

[105] Chia X., Eng A.Y.S., Ambrosi A., Tan S.M., Pumera M. (2015). Electrochemistry of nanostructured layered transition-metal dichalcogenides. Chem. Rev..

[106] Tsai C., Chan K., Abild-Pedersen F., Nørskov J.K. (2014). Active edge sites in MoSe2 and WSe2 catalysts for the hydrogen evolution reaction: A density functional study. Phys. Chem. Chem. Phys..

[107] Coleman J.N., Lotya M., O’Neill A., Bergin S.D., King P.J., Khan U., Young K., Gaucher A., De S., Smith R.J. (2011). Two-dimensional nanosheets produced by liquid exfoliation of layered materials. Science.

[108] Nicolosi V., Chhowalla M., Kanatzidis M.G., Strano M.S., Coleman J.N. (2013). Liquid exfoliation of layered materials. Science.

[109] Lee K., Kim H.-Y., Lotya M., Coleman J.N., Kim G.-T., Duesberg G.S. (2011). Electrical characteristics of molybdenum disulfide flakes produced by liquid exfoliation. Adv. Mater..

[110] Chhowalla M., Liu Z., Zhang H. (2015). Two-dimensional transition metal dichalcogenide (TMD) nanosheets. Chem. Soc. Rev..

[111] Mayorga-Martinez C.C., Khezri B., Eng A.Y.S., Sofer Z., Ulbrich P., Pumera M. (2016). Bipolar Electrochemical Synthesis of WS2 Nanoparticles and Their Application in Magneto-Immunosandwich Assay. Adv. Funct. Mater..

[112] Toh R.J., Mayorga-Martinez C.C., Sofer Z., Pumera M. (2016). MoSe2 nanolabels for electrochemical immunoassays. Anal. Chem..

[113] Mazanek V., Mayorga-Martinez C.C., Bousa D., Sofer Z., Pumera M. (2018). WSe2 nanoparticles with enhanced hydrogen evolution reaction prepared by bipolar electrochemistry: Application in competitive magneto-immunoassay. Nanoscale.

[114] Mayorga-Martinez C.C., Latiff N.M., Eng A.Y.S., Sofer Z., Pumera M. (2016). Black phosphorus nanoparticle labels for immunoassays via hydrogen evolution reaction mediation. Anal. Chem..

[115] Sofer Z., Bouša D., Luxa J., Mazanek V., Pumera M. (2016). Few-layer black phosphorus nanoparticles. Chem. Commun..

[116] Li D., Castillo A.E.D.R., Jussila H., Ye G., Ren Z., Bai J., Chen X., Lipsanen H., Sun Z., Bonaccorso F. (2016). Black phosphorus polycarbonate polymer composite for pulsed fibre lasers. Appl. Mater. Today.

[117] Cheng W., Compton R.G. (2014). Electrochemical detection of nanoparticles by ‘nano-impact’methods. TrAC Trends Anal. Chem..

[118] Wood J.D., Wells S.A., Jariwala D., Chen K.-S., Cho E., Sangwan V.K., Liu X., Lauhon L.J., Marks T.J., Hersam M.C. (2014). Effective passivation of exfoliated black phosphorus transistors against ambient degradation. Nano Lett..

[119] Kwon S.J., Bard A.J. (2012). DNA analysis by application of Pt nanoparticle electrochemical amplification with single label response. J. Am. Chem. Soc..

[120] Khoshfetrat S.M., Khoshsafar H., Afkhami A., Mehrgardi M.A., Bagheri H. (2019). Enhanced visual wireless electrochemiluminescence immunosensing of prostate-specific antigen based on the luminol loaded into MIL-53 (Fe)-NH2 accelerator and hydrogen evolution reaction mediation. Anal. Chem..

[121] Khoshfetrat S.M., Dorraji P.S., Shayan M., Khatami F., Omidfar K. (2022). Smartphone-based electrochemiluminescence for visual simultaneous detection of RASSF1A and SLC5A8 tumor suppressor gene methylation in thyroid cancer patient plasma. Anal. Chem..

[122] Ino K., Yaegaki R., Hiramoto K., Nashimoto Y., Shiku H. (2020). Closed bipolar electrode array for on-chip analysis of cellular respiration by cell aggregates. ACS Sens..

[123] Wu M.-S., Yuan D.-J., Xu J.-J., Chen H.-Y. (2013). Sensitive electrochemiluminescence biosensor based on Au-ITO hybrid bipolar electrode amplification system for cell surface protein detection. Anal. Chem..

[124] Khoshfetrat S.M., Bagheri H., Mehrgardi M.A. (2018). Visual electrochemiluminescence biosensing of aflatoxin M1 based on luminol-functionalized, silver nanoparticle-decorated graphene oxide. Biosens. Bioelectron..

[125] Huang Y., Wen Q., Jiang J.-H., Shen G.-L., Yu R.-Q. (2008). A novel electrochemical immunosensor based on hydrogen evolution inhibition by enzymatic copper deposition on platinum nanoparticle-modified electrode. Biosens. Bioelectron..

[126] Chen H., Jiang J.-H., Li Y.-F., Deng T., Shen G.-L., Yu R.-Q. (2007). A novel piezoelectric immunoagglutination assay technique with antibody-modified liposome. Biosens. Bioelectron..

[127] Chen Z.-P., Peng Z.-F., Luo Y., Qu B., Jiang J.-H., Zhang X.-B., Shen G.-L., Yu R.-Q. (2007). Successively amplified electrochemical immunoassay based on biocatalytic deposition of silver nanoparticles and silver enhancement. Biosens. Bioelectron..

[128] Katz E., Alfonta L., Willner I. (2001). Chronopotentiometry and faradaic impedance spectroscopy as methods for signal transduction in immunosensors. Sens. Actuators B Chem..

[129] Sharma A., Rao V.K., Kamboj D.V., Jain R. (2014). Electrochemical Immunosensor for Staphylococcal Enterotoxin B (SEB) Based on Platinum Nanoparticles-Modified Electrode Using Hydrogen Evolution Inhibition Approach. Electroanalysis.

[130] Zhang J., Ting B.P., Khan M., Pearce M.C., Yang Y., Gao Z., Ying J.Y. (2010). Pt nanoparticle label-mediated deposition of Pt catalyst for ultrasensitive electrochemical immunosensors. Biosens. Bioelectron..

[131] Que X., Chen X., Fu L., Lai W., Zhuang J., Chen G., Tang D. (2013). Platinum-catalyzed hydrogen evolution reaction for sensitive electrochemical immunoassay of tetracycline residues. J. Electroanal. Chem..

[132] Liao K.-T., Huang H.-J. (2005). Femtomolar immunoassay based on coupling gold nanoparticle enlargement with square wave stripping voltammetry. Anal. Chim. Acta.

[133] Chu X., Xiang Z.-F., Fu X., Wang S.-P., Shen G.-L., Yu R.-Q. (2005). Silver-enhanced colloidal gold metalloimmunoassay for Schistosoma japonicum antibody detection. J. Immunol. Methods.

[134] Spain E., Gilgunn S., Sharma S., Adamson K., Carthy E., O’Kennedy R., Forster R.J. (2016). Detection of prostate specific antigen based on electrocatalytic platinum nanoparticles conjugated to a recombinant scFv antibody. Biosens. Bioelectron..

[135] Maltez-da Costa M., De La Escosura-Muñiz A., Merkoçi A. (2010). Electrochemical quantification of gold nanoparticles based on their catalytic properties toward hydrogen formation: Application in magnetoimmunoassays. Electrochem. Commun..

[136] de la Escosura-Muñiz A., Maltez-da Costa M., Sánchez-Espinel C., Díaz-Freitas B., Fernández-Suarez J., González-Fernández Á., Merkoçi A. (2010). Gold nanoparticle-based electrochemical magnetoimmunosensor for rapid detection of anti-hepatitis b virus antibodies in human serum. Biosens. Bioelectron..

[137] de la Escosura-Muniz A., Sanchez-Espinel C., Díaz-Freitas B., Gonzalez-Fernandez A., Maltez-da Costa M., Merkoci A. (2009). Rapid identification and quantification of tumor cells using an electrocatalytic method based on gold nanoparticles. Anal. Chem..

[138] Maltez-da Costa M., De La Escosura-Muñiz A., Nogués C., Barrios L., Ibáñez E., Merkoçi A. (2012). Simple monitoring of cancer cells using nanoparticles. Nano Lett..

[139] Boya M., Ozkaya-Ahmadov T., Swain B.E., Chu C.-H., Asmare N., Civelekoglu O., Liu R., Lee D., Tobia S., Biliya S. (2022). High throughput, label-free isolation of circulating tumor cell clusters in meshed microwells. Nat. Commun..

[140] Bednarz-Knoll N., Alix-Panabières C., Pantel K. (2011). Clinical relevance and biology of circulating tumor cells. Breast Cancer Res..

[141] Perfézou M., Turner A., Merkoçi A. (2012). Cancer detection using nanoparticle-based sensors. Chem. Soc. Rev..

[142] Cohen S.J., Alpaugh R.K., Gross S., O’Hara S.M., Smirnov D.A., Terstappen L.W.M.M., Allard W.J., Bilbee M., Cheng J.D., Hoffman J.P. (2006). Isolation and characterization of circulating tumor cells in patients with metastatic colorectal cancer. Clin. Color. Cancer.

[143] Riethdorf S., Fritsche H., Mueller V., Rau T., Schindlbeck C., Rack B., Janni W., Coith C., Beck K., Jaenicke F. (2007). Detection of circulating tumor cells in peripheral blood of patients with metastatic breast cancer: A validation study of the cellsearch system. Clin. Cancer Res..

[144] Maltez-da Costa M., de la Escosura-Muñiz A., Nogués C., Barrios L., Ibáñez E., Merkoçi A. (2012). Detection of circulating cancer cells using electrocatalytic gold nanoparticles. Small.

[145] de la Escosura-Muñiz A., Baptista-Pires L., Serrano L., Altet L., Francino O., Sánchez A., Merkoçi A. (2016). Magnetic bead/gold nanoparticle double-labeled primers for electrochemical detection of isothermal amplified leishmania dna. Small.

[146] Hassan A.-R.H.A.-A., de la Escosura-Muñiz A., Merkoçi A. (2015). Highly sensitive and rapid determination of escherichia coli o157:h7 in minced beef and water using electrocatalytic gold nanoparticle tags. Biosens. Bioelectron..

[147] Sun A.-L. (2015). Sensitive electrochemical immunoassay with signal enhancement based on nanogold-encapsulated poly (amidoamine) dendrimer-stimulated hydrogen evolution reaction. Analyst.

[148] Mayorga-Martinez C.C., Chamorro-Garcia A., Merkoçi A. (2015). Electrochemical impedance spectroscopy (bio) sensing through hydrogen evolution reaction induced by gold nanoparticles. Biosens. Bioelectron..

[149] De La Escosura-Muñiz A., Plichta Z., Horák D., Merkoçi A. (2015). Alzheimer’s disease biomarkers detection in human samples by efficient capturing through porous magnetic microspheres and labelling with electrocatalytic gold nanoparticles. Biosens. Bioelectron..

[150] Sun K., Xu W., Lin X., Tian S., Lin W., Zhou D., Sun X. (2021). Electrochemical oxygen reduction to hydrogen peroxide via a two-electron transfer pathway on carbon-based single-atom catalysts. Adv. Mater. Interfaces.

[151] Wu J., Tang J., Dai Z., Yan F., Ju H., Murr N.E. (2006). A disposable electrochemical immunosensor for flow injection immunoassay of carcinoembryonic antigen. Biosens. Bioelectron..

[152] Cui R., Huang H., Yin Z., Gao D., Zhu J.-J. (2008). Horseradish peroxidase-functionalized gold nanoparticle label for amplified immunoanalysis based on gold nanoparticles/carbon nanotubes hybrids modified biosensor. Biosens. Bioelectron..

[153] Daneshpour M., Syed moradi L., Izadi P., Omidfar K. (2016). Femtomolar level detection of rassf1a tumor suppressor gene methylation by electrochemical nano-genosensor based on fe3o4/tmc/au nanocomposite and pt-modified electrode. Biosens. Bioelectron..

[154] Fan G.-C., Gu S., Zhang D., Hu Z., Luo X. (2020). Platinum-based nanocomposite as oxygen reduction catalyst for efficient signal amplification: Toward building of high-performance photocathodic immunoassay. Biosens. Bioelectron..

[155] Xu Q., Wang L., Lei J., Deng S., Ju H. (2013). Platinum nanodendrite functionalized graphene nanosheets as a non-enzymatic label for electrochemical immunosensing. J. Mater. Chem. B.

[156] Leng C., Wu J., Xu Q., Lai G., Ju H., Yan F. (2011). A highly sensitive disposable immunosensor through direct electro-reduction of oxygen catalyzed by palladium nanoparticle decorated carbon nanotube label. Biosens. Bioelectron..

[157] Deng S., Lei J., Huang Y., Yao X., Ding L., Ju H. (2012). Electrocatalytic reduction of coreactant by highly loaded dendrimer-encapsulated palladium nanoparticles for sensitive electrochemiluminescent immunoassay. Chem. Commun..

[158] Zhu C., Du D., Eychmueller A., Lin Y. (2015). Engineering ordered and nonordered porous noble metal nanostructures: Synthesis, assembly, and their applications in electrochemistry. Chem. Rev..

[159] Zhang C., Liu W., Li Z., Yan B., Lin J., Chen C., Zhang L., Lu Y. (2022). Accelerated mimetic oxidase activity of polydopamine-dressed PdCu nanozyme for the detection of ascorbic acid related bioenzymes. ACS Sustain. Chem. Eng..

[160] He W., Wu X., Liu J., Hu X., Zhang K., Hou S., Zhou W., Xie S. (2010). Design of AgM bimetallic alloy nanostructures (M = Au, Pd, Pt) with tunable morphology and peroxidase-like activity. Chem. Mater..

[161] Cao X., Han Y., Gao C., Huang X., Xu Y., Wang N. (2013). PtAg nanowires: Facile synthesis and their applications as excellent oxygen reduction electrocatalysts for label-free electrochemical immunoassay. J. Mater. Chem. A.

[162] Ge X.-Y., Feng Y.-G., Cen S.-Y., Wang A.-J., Mei L.-P., Luo X., Feng J.-J. (2021). A label-free electrochemical immnunosensor based on signal magnification of oxygen reduction reaction catalyzed by uniform PtCo nanodendrites for highly sensitive detection of carbohydrate antigen 15-3. Anal. Chim. Acta.

[163] Zhang J., Xu X., Qiang Y. (2020). Ultrasensitive electrochemical aptasensor for ochratoxin A detection using AgPt bimetallic nanoparticles decorated iron-porphyrinic metal-organic framework for signal amplification. Sens. Actuators B Chem..

[164] Yang T.-H., Ahn J., Shi S., Wang P., Gao R., Qin D. (2020). Noble-metal nanoframes and their catalytic applications. Chem. Rev..

[165] Wang X., Ruditskiy A., Xia Y. (2016). Rational design and synthesis of noble-metal nanoframes for catalytic and photonic applications. Natl. Sci. Rev..

[166] Yoo S., Kim J., Choi S., Park D., Park S. (2019). Two-dimensional nanoframes with dual rims. Nat. Commun..

[167] Fang Z., Wang Y., Liu C., Chen S., Sang W., Wang C., Zeng J. (2015). Rational design of metal nanoframes for catalysis and plasmonics. Small.

[168] Wang A.-J., Zhu X.-Y., Chen Y., Yuan P.-X., Luo X., Feng J.-J. (2019). A label-free electrochemical immunosensor based on rhombic dodecahedral Cu3Pt nanoframes with advanced oxygen reduction performance for highly sensitive alpha-fetoprotein detection. Sens. Actuators B. Chem..

[169] Zhang K., Dong H., Dai W., Meng X., Lu H., Wu T., Zhang X. (2017). Fabricating Pt/Sn–In_2_O_3_ nanoflower with advanced oxygen reduction reaction performance for high-sensitivity microRNA electrochemical detection. Anal. Chem..

[170] Li L., Liu X., Yang L., Zhang S., Zheng H., Tang Y., Wong D.K.Y. (2019). Amplified oxygen reduction signal at a Pt-Sn-modified TiO_2_ nanocomposite on an electrochemical aptasensor. Biosens. Bioelectron..

[171] Huo X., Liu P., Zhu J., Liu X., Ju H. (2016). Electrochemical immunosensor constructed using TiO_2_ nanotubes as immobilization scaffold and tracing tag. Biosens. Bioelectron..

[172] Liu X., Huo X., Liu P., Tang Y., Xu J., Ju H. (2017). TiO_2_ nanowire arrays modified with a simultaneous “etching, doping and deposition” technique for ultrasensitive amperometric immunosensing. Biosens. Bioelectron..

[173] Wang R., Wang A.-J., Liu W.-D., Yuan P.-X., Xue Y., Luo X., Feng J.-J. (2018). A novel label-free electrochemical immunosensor for ultra-sensitively detecting prostate specific antigen based on the enhanced catalytic currents of oxygen reduction catalyzed by core-shell au@pt nanocrystals. Biosens. Bioelectron..

[174] Toyos-Rodríguez C., Adawy A., García-Alonso F.J., Alfredo de la Escosura-Muniz A. (2022). Enhancing the electrocatalytic activity of palladium nanocluster tags by selective introduction of gold atoms: Application for a wound infection biomarker detection. Biosens. Bioelectron..

[175] Wang R., Feng J.-J., Liu W.-D., Jiang L.-Y., Wang A.-J. (2017). A novel label-free electrochemical immunosensor based on the enhanced catalytic currents of oxygen reduction by AuAg hollow nanocrystals for detecting carbohydrate antigen 199. Biosens. Bioelectron..

[176] Zhou Y., Chai Y., Yuan R. (2019). Highly efficient dual-polar electrochemiluminescence from Au25 nanoclusters: The next generation of multibiomarker detection in a single step. Anal. Chem..

[177] Polsky R., Harper J.C., Wheeler D.R., Dirk S.M., Rawlings J.A., Brozik S.M. (2007). Reagentless electrochemical immunoassay using electrocatalytic nanoparticle-modified antibodies. Chem. Commun..

[178] Liu X., Zhang Y., Lei J., Xue Y., Cheng L., Ju H. (2010). Quantum dots based electrochemiluminescent immunosensor by coupling enzymatic amplification with self-produced coreactant from oxygen reduction. Anal. Chem..

[179] Deng S., Lei J., Huang Y., Cheng Y., Ju H. (2013). Electrochemiluminescent quenching of quantum dots for ultrasensitive immunoassay through oxygen reduction catalyzed by nitrogen-doped graphene-supported hemin. Anal. Chem..

[180] Gong K., Du F., Xia Z., Durstock M., Dai L. (2009). Nitrogen-doped carbon nanotube arrays with high electrocatalytic activity for oxygen reduction. Science.

[181] Tang Y., Allen B.L., Kauffman D.R., Star A. (2009). Electrocatalytic activity of nitrogen-doped carbon nanotube cups. J. Am. Chem. Soc..

[182] Mani V., Chikkaveeraiah B.V., Patel V., Gutkind J.S., Rusling J.F. (2009). Ultrasensitive immunosensor for cancer biomarker proteins using gold nanoparticle film electrodes and multienzyme-particle amplification. ACS Nano.

[183] Deng S., Hou Z., Lei J., Lin D., Hu Z., Yan F., Ju H. (2011). Signal amplification by adsorption-induced catalytic reduction of dissolved oxygen on nitrogen-doped carbon nanotubes for electrochemiluminescent immunoassay. Chem. Commun..

[184] Luo X., Luo Z., Li S., Fang Q., Xu W., Wang H., Wang Y., Bao G.-M., Gu W., Zhu C. (2023). Nanozymatic biofuel cell-enabled self-powered sensing system for a sensitive immunoassay. Anal. Chem..

[185] Luo X., Yang M., Song W., Fang Q., Wei X., Jiao L., Xu W., Kang Y., Wang H., Wu N. (2021). Neutral zn-air battery assembled with single-atom iridium catalysts for sensitive self-powered sensing system. Adv. Funct. Mater..

[186] Zhou Y., Fan H.J. (2020). Progress and challenge of amorphous catalysts for electrochemical water splitting. ACS Mater. Lett..

[187] Wang P., Wang B. (2021). Designing self-supported electrocatalysts for electrochemical water splitting: Surface/interface engineering toward enhanced electrocatalytic performance. ACS Appl. Mater. Interfaces.

[188] Rivas L., de la Escosura-Muñiz A., Pons J., Merkoçi A. (2014). Alzheimer disease biomarker detection through electrocatalytic water oxidation induced by iridium oxide nanoparticles. Electroanalysis.

[189] Quesada-González D., Baiocco A., Martos A.A., De La Escosura-Muñiz A., Palleschi G., Merkoçi A. (2019). Iridium oxide (IV) nanoparticle-based electrocatalytic detection of PBDE. Biosens. Bioelectron..

[190] Han Y., Qiu Z., Nawale G.N., Varghese O.P., Hilborn J., Tian B., Leifer K. (2019). MicroRNA detection based on duplex-specific nuclease-assisted target recycling and gold nanoparticle/graphene oxide nanocomposite-mediated electrocatalytic amplification. Biosens. Bioelectron..

[191] Deng D., Hao Y., Yang S., Han Q., Liu L., Xiang Y., Tu F., Xia N. (2019). A signal-on electrochemical biosensor for evaluation of caspase-3 activity and cell apoptosis by the generation of molecular electrocatalysts on graphene electrode surface for water oxidation. Sens. Actuators B Chem..

[192] Xia N., Deng D., Yang S., Hao Y., Wang L., Liu Y., An C., Han Q., Liu L. (2019). Electrochemical immunosensors with protease as the signal label for the generation of peptide-Cu (II) complexes as the electrocatalysts toward water oxidation. Sens. Actuators B Chem..

[193] Tian J., Cheng N., Liu Q., Xing W., Sun X. (2015). Cobalt phosphide nanowires: Efficient nanostructures for fluorescence sensing of biomolecules and photocatalytic evolution of dihydrogen from water under visible light. Angew. Chem..

[194] Zhu X., Yuan S., Ju Y., Yang J., Zhao C., Liu H. (2019). Water splitting-assisted electrocatalytic oxidation of glucose with a metal-organic framework for wearable nonenzymatic perspiration sensing. Anal. Chem..

[195] Chen Q., Ranaweera R., Luo L. (2018). Hydrogen bubble formation at hydrogen-insertion electrodes. J. Phys. Chem. C.

[196] Zlotea C., Campesi R., Cuevas F., Leroy E., Dibandjo P., Volkringer C., Loiseau T., Férey G., Latroche M. (2010). Pd nanoparticles embedded into a metal-organic framework: Synthesis, structural characteristics, and hydrogen sorption properties. J. Am. Chem. Soc..

[197] Cheon Y.E., Suh M.P. (2009). Enhanced hydrogen storage by palladium nanoparticles fabricated in a redox-active metal-organic framework. Angew. Chem..

[198] Chinnappan R., Ramachandran L., Uttam I., Citartan M., Ballal N.V., Mani N.K. (2025). From biomarkers to biosensors: Modern approaches for the detection of matrix metalloproteinases (MMPs). Biosensors.

[199] Iglesias-Mayor A., Amor-Gutiérrez O., Costa-García A., de la Escosura-Muñiz A. (2019). Nanoparticles as emerging labels in electrochemical immunosensors. Sensors.

[200] Domínguez-Aragón A., Zaragoza-Contreras E.A., Figueroa-Miranda G., Offenhäusser A., Mayer D. (2022). Electrochemical immunosensor using electroactive carbon nanohorns for signal amplification for the rapid detection of carcinoembryonic antigen. Biosensors.

[201] Wang M., Gao F., Ni S., Song Y., Wang C., Li Q., Zhao P. (2023). Electrochemical immunosensor based on gold–thionine for detection of subarachnoid hemorrhage biomarker. Front. Bioeng. Biotechnol..

[202] GeGen S., Meng G., Aodeng G., Ga L., Ai J. (2025). Advances in aptamer-based electrochemical biosensors for disease diagnosis: Integration of DNA and nanomaterials. Nanoscale Horizons.

[203] Chen C., La M., Yi X., Huang M., Xia N., Zhou Y. (2023). Progress in electrochemical immunosensors with alkaline phosphatase as the signal label. Biosensors.

[204] du Plooy J., Jahed N., Iwuoha E., Pokpas K. (2023). Advances in paper-based electrochemical immunosensors: Review of fabrication strategies and biomedical applications. R. Soc. Open Sci..

[205] Bucur B., Purcarea C., Andreescu S., Vasilescu A. (2021). Addressing the selectivity of enzyme biosensors: Solutions and perspectives. Sensors.

[206] Ochoa-Ruiz A.G., Parra G., López-Espinoza D., Astudillo P., Galyamin D., Sabaté N., Esquivel J.P., Vallejo-Cardona A.A. (2023). Electrochemical immunosensors: The evolution from ELISA to EμPADs. Electroanalysis.

[207] Balbaied T., Moore E. (2019). Overview of optical and electrochemical alkaline phosphatase (ALP) biosensors: Recent approaches in cell culture techniques. Biosensors.

